# Development of a proactive-diagnostic smart packaging system for real-time freshness monitoring and shelf-life extension of refrigerated broiler breast

**DOI:** 10.1016/j.psj.2026.107532

**Published:** 2026-07-30

**Authors:** Krittanon Chotprom, Somchart Tana, Trakul Prommajak, Kunakorn Katsri, Napatsorn Montha, Phatthawin Setthaya, Piyanart Nuntalit, Thanaporn Bunmee

**Affiliations:** a Division of Animal Science, School of Agriculture and Natural Resources, University of Phayao, 56000, Thailand; b Division of Food Safety, School of Agriculture and Natural Resources, University of Phayao, 56000, Thailand; c Division of Food Science and Technology, School of Agriculture and Natural Resources, University of Phayao, 56000, Thailand; d Department of Animal and Aquatic Science, Faculty of Agriculture, Chiang Mai University, Chiang Mai, 50200, Thailand; e Division of Product Development Technology, Faculty of Agro-Industry, Chiang Mai University, Chiang Mai, 50100, Thailand; f Animal Science, Nakhon Si Thammarat College of Agriculture and Technology, Nakhon Si Thammarat, Thailand

**Keywords:** Smart packaging, Broiler breast, Lemongrass essential oil, Butterfly pea anthocyanin, Shelf-life

## Abstract

Fresh broiler breast meat is highly susceptible to rapid microbial proliferation and lipid oxidation during refrigerated storage, necessitating advanced preservation and quality-monitoring strategies. This study developed an eco-friendly, dual-functional “proactive-diagnostic” smart packaging system by incorporating lemongrass essential oil (LEO; 0, 2.5, and 5.0% w/v) into a transglutaminase-crosslinked fish gelatin/sodium alginate active matrix, coupled with a headspace butterfly pea anthocyanin (BPA) colorimetric indicator. Results indicated that LEO incorporation significantly (*p* < 0.05) enhanced the functional properties of the films, notably increasing UV-shielding capacity and reduced water solubility by 26%, thereby confirming improved structural stability within high-moisture packaging environments. Mechanical analysis demonstrated a concentration-dependent plasticizing effect of LEO, which significantly improved film elongation at break while maintaining adequate tensile strength. In application trials with fresh broiler breasts stored at 4 ± 2°C, the 5.0% LEO-loaded active film exhibited superior antimicrobial and antioxidant performance, effectively suppressing *Escherichia coli* and *Staphylococcus aureus* growth. By day 7 of storage, the LEO-loaded active film maintained total volatile basic nitrogen (TVB-N) and TBARS values at 15.77 ± 0.35 mg N/100 g and 1.32 ± 0.10 mg MDA/kg, respectively, remaining well below established microbiological spoilage thresholds. Furthermore, the LEO-loaded film substantially minimized drip loss and stabilized surface redness (a*), thereby preserving myofibrillar structural integrity and visual acceptability. Concurrently, the headspace BPA indicator exhibited a distinct, non-destructive halochromic transition from dark purple to blue and green, driven by the structural transformation of anthocyanins into chalcone derivatives in response to muscle pH shifts and TVB-N accumulation. In conclusion, this 5.0% LEO-loaded smart packaging system extends the refrigerated shelf-life of broiler breast by at least 3 days and provides a reliable, non-destructive tool for real-time quality validation, offering a potential sustainable approach to reduce food waste in the poultry supply chain.

## Highlights

• Active film extends refrigerated broiler breast shelf-life by 3 days.
• Gelatin/alginate film with lemongrass oil exhibits strong antimicrobial activity.
• Anthocyanin indicators enable real-time visual monitoring of poultry freshness.
• Color shifts correlate strongly with TVB-N and pH increases in broiler meat.
• The smart packaging system potentially reduces food loss in the poultry chain.

## Introduction

The poultry industry faces significant challenges in maintaining the quality and safety of fresh broiler meat, which is inherently susceptible to rapid spoilage. Due to its high moisture content and rich nutrient profile, broiler breast meat undergoes accelerated microbial proliferation and lipid oxidation during refrigerated storage (Ahmad et al., 2026). Unlike red meat, broiler breast muscle contains a higher proportion of polyunsaturated fatty acids (PUFAs) which, when combined with the presence of prooxidative heme proteins, renders it exceptionally vulnerable to lipid peroxidation and the subsequent development of rancidity. These biochemical processes lead to the accumulation of off-flavors, discoloration, and the evolution of volatile compounds such as total volatile basic nitrogen (TVB-N), ultimately resulting in substantial economic losses and food waste (Ahmed et al., 2022). Integrating biobased matrices with active functionalities not only addresses UN Sustainable Development Goal (SDG) Target 12.3 by reducing post-slaughter losses but also mitigates the environmental burden of conventional plastic waste, aligning with SDG 13 (Climate Action) to reduce the carbon footprint of the poultry supply chain (Barone and Aschemann-Witzel, 2022; Bhargava et al., 2020). Consequently, transitioning from synthetic plastics to biodegradable, multifunctional packaging is a promising approach for a sustainable and resilient poultry production system.

Multifunctional biobased smart films represent a developing technology category that includes active and intelligent systems. Active packaging interacts directly with the food or its environment to extend shelf-life, often through the release of antimicrobial or antioxidant agents (Domínguez et al., 2018). In this context, Lemongrass Essential Oil (LEO; *Cymbopogon citratus*) serves as a potent active agent. Rich in citral (geranial and neral), LEO exerts strong antimicrobial activity by disrupting the cell membranes of key meat spoilage bacteria, such as *Salmonella* Heidelberg, *Pseudomonas* spp., and *Staphylococcus aureus* (Aiemsaard et al., 2011; Cui et al., 2017; Liu et al., 2020; Syahida et al., 2021; Rabbani et al., 2026). Despite its potent antioxidant and antimicrobial properties, the industrial application of LEO is often hindered by its high volatility and the potential to impart strong off-flavors to food matrices (Ben-Fadhel et al., 2024). To address these challenges, encapsulation within the polymeric matrix emerges as a strategic approach. By incorporating LEO into a stabilized emulsion, the interfacial surface area is maximized, thereby enhancing its functional potency and bioavailability (do Nascimento et al., 2023). However, the efficacy of these nanodroplets is highly dependent on the carrier matrix. To achieve this, a synergistic gelatin/alginate network, cross-linked with transglutaminase, is proposed to act as a robust scaffold. This specific polymeric lattice is hypothesized to provide a 'tortuous path' that anchors the emulsified LEO droplets, shielding sensitive constituents like citral from environmental degradation while ensuring a controlled release (Kocatepe et al., 2025). Such an integrated system not only stabilizes the bioactives but also modulates their sensory impact, potentially offering a superior biobased alternative to synthetic packaging for poultry preservation.

Simultaneously, intelligent packaging provides real-time "diagnostics" by monitoring freshness markers. During spoilage, the pH of broiler meat shifts as microorganisms break down proteins into alkaline nitrogenous compounds (Lee et al., 2024). Butterfly pea anthocyanins (BPA) act as intelligent colorimetric sensors that exhibit visibly color change in response to pH shifts caused by food decay (Boonsiriwit et al., 2021; Solak et al., 2023). Positioning these indicators within the package headspace facilitates real-time, nondestructive monitoring of broiler freshness, allowing for quality verification without compromising the package seal or cold chain stability (Kanatt, 2020; Xu et al., 2025). Despite recent advancements in standalone active or intelligent films, research on a synchronized, "proactive-diagnostic" system that simultaneously extends shelf-life via controlled vapor release and non-destructively tracks real-time spoilage kinetics in the package headspace remains remarkably scarce, particularly for highly perishable poultry matrices.

Therefore, the objective of this study was to develop and comprehensively evaluate an eco-friendly, dual-functional smart packaging system engineered for refrigerated broiler breast meat preservation and quality monitoring. It was hypothesized that the controlled release of LEO from the cross-linked matrix would suppress targeted microbial and oxidative spoilage, while the volatile nitrogen compounds in the headspace would trigger distinct, measurable colorimetric shifts in the BPA indicator. To verify these effects, an active biopolymer matrix comprising transglutaminase-crosslinked fish gelatin and sodium alginate was formulated with emulsified LEO at varying concentrations (0, 2.5, and 5.0% w/v). The film's structural integrity, mechanical properties (tensile strength and elongation at break), and soil biodegradability profiles were systematically characterized. Concurrently, an optimized BPA indicator was positioned within the packaging headspace to monitor fresh broiler breast meat stored at 4 ± 2 for 12 days. The efficacy of the active film in retarding lipid oxidation (thiobarbituric acid reactive substances; TBARS), protein degradation (TVB-N), and the proliferation of specific spoilage bacteria (*Pseudomonas* spp., *Escherichia coli*, and total viable counts) was rigorously quantified. Crucially, the colorimetric kinetics ($L^{*},a^{*},b^{*}$, and ΔE) of the BPA indicators were tracked. Finally, Principal Component Analysis (PCA) was employed to establish a precise mathematical correlation between the quantitative colorimetric shifts of the indicator and the actual meat spoilage biomarkers. This work offers a sustainable, highly transparent, and scientifically validated smart packaging solution to mitigate food waste and enhance safety in the poultry supply chain. The overall experimental framework of this study is schematically illustrated in Fig. 1.

**Fig. 1:**
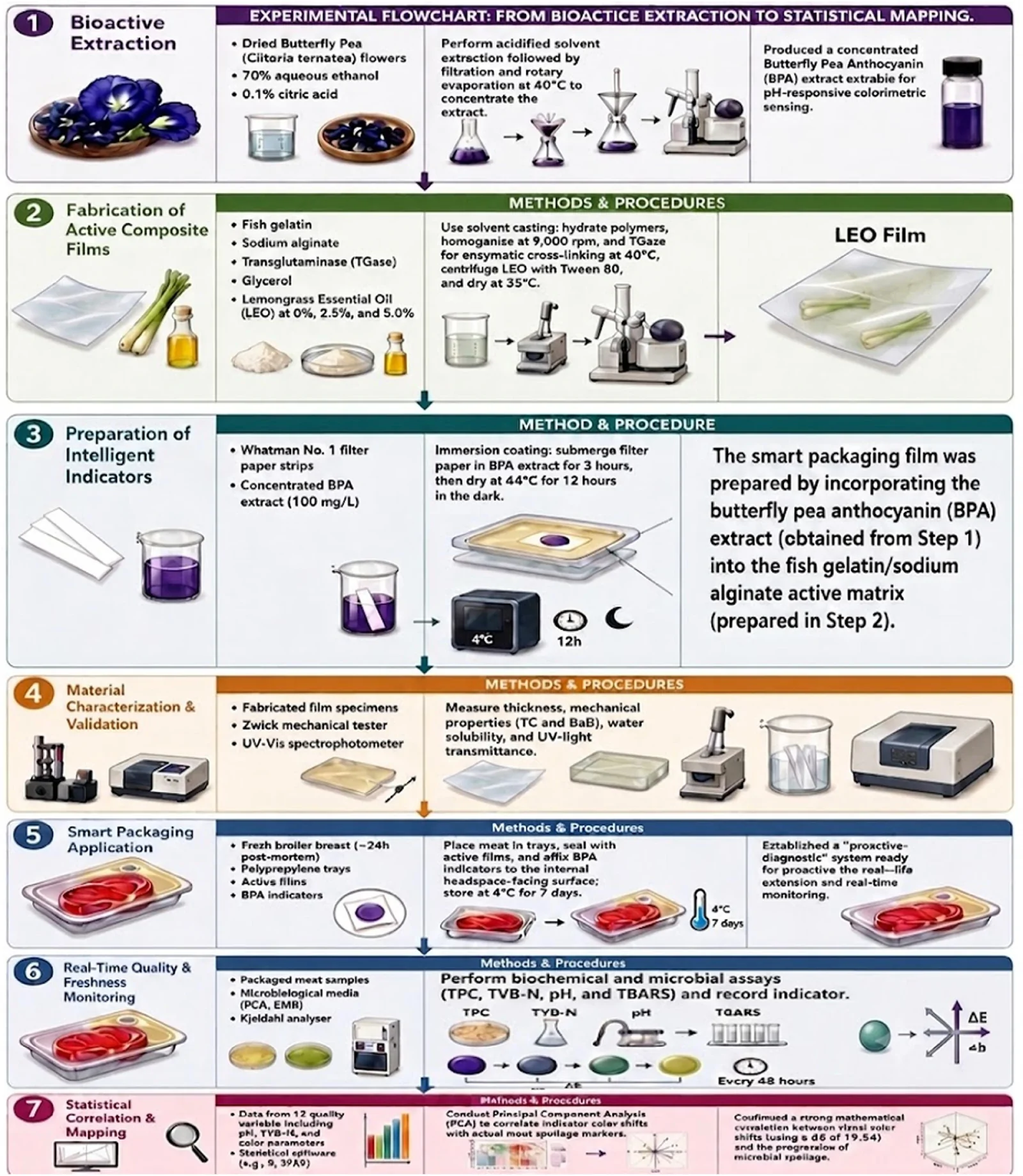
Schematic illustration of the overall experimental design. The framework outlines the extraction of bioactive compounds from butterfly pea flowers, the fabrication of smart bio-composite films incorporated with lemongrass essential oil (LEO), the preparation of intelligent indicators, and their application for real-time quality and freshness monitoring of packaged broiler breast meat.

## Materials and methods

### Materials and reagents

Dried Butterfly Pea (*Clitoria ternatea* L.) flowers were obtained from an organic farm in Phayao, Thailand, ground, and sieved through a 60-mesh screen. Lemongrass essential oil (LEO) was purchased from Botanic Essence (Bangkok, Thailand). Fish gelatin (250 Bloom) and sodium alginate were purchased from Union Science Co., Ltd. (Chiang Mai, Thailand). Transglutaminase (TGase, 100 U/g) was provided by Ajinomoto (Thailand) Co., Ltd. All analytical grade reagents, including thiobarbituric acid (TBA), trichloroacetic acid (TCA), and 1,1,3,3-tetramethoxypropane (TMP), were sourced from Sigma-Aldrich and Merck. Microbiological media (PCA, EMB, and Baird-Parker agar) were obtained from Oxoid (UK). Fresh broiler breasts (∼24 h postmortem) were procured from a local slaughterhouse and transported to the laboratory at 4 ± 1°C within 30 min. Since the broiler breast meat samples were obtained from a commercial slaughterhouse operating under standard industry practices, specific animal welfare approval was not required for the laboratory phase. However, the study protocol was reviewed and approved by the Institutional Animal Care and Use Committee (IACUC) of the University of Phayao, Thailand (Approval No. 2-009-68)

### Preparation of colorimetric freshness indicators

The extraction of anthocyanins from butterfly pea (*Clitoria ternatea* L.) flowers was performed using an acidified solvent extraction method according to Gao et al. (2023) with modifications. Briefly, dried flower powder was immersed in a 70% (v/v) aqueous ethanol solution acidified with 0.1% (v/v) citric acid to maintain a pH below 3.0, utilizing a solid-to-liquid ratio of 1:20 (w/v). The mixture was continuously agitated using a magnetic stirrer at 250 rpm for 24 h at room temperature (25± 2°C) under complete darkness to optimize extraction efficiency. The resulting extract was filtered through Whatman No. 1 filter paper and subsequently concentrated using a rotary evaporator at 40°C under reduced pressure (Büchi, Flawil, Switzerland) until a viscous crude extract was obtained. The concentrated extract was lyophilized to obtain a stable powder and stored at −20°C. To prepare the colorimetric indicators, the lyophilized BPA extract was redissolved in distilled water to achieve a precise concentration of 100 mg/L. Whatman No. 1 filter paper strips (dimensions: 2 cm x 2 cm) were completely submerged in the BPA solution at a ratio of 10 strips per 50 mL of solution for 3 h at room temperature. The loaded strips were then removed and transferred to a dark, forced-air oven to dry at 40°C for 12 h to minimize photo-oxidation and achieve uniform moisture removal. The dried smart indicators were finally stored in light-protected, vacuum-sealed desiccators at 4°C prior to integration into the poultry packaging system to prevent premature oxidation and environmental moisture absorption.

### Preparation of LEO-loaded gelatin/alginate composite films

The composite films were prepared using a modified solvent casting method. Briefly, 1.7 g of fish gelatin was hydrated in 200 mL of distilled water at 65°C for 20 min. After cooling to 25 ± 2°C, 4 g of sodium alginate was incorporated and homogenized at 9,000 rpm for 15 min using an IKA Ultra-Turrax T25 homogenizer (IKA Werke GmbH and Co. KG, Staufen, Germany). Glycerol (8 g) and TGase (0.017 g; 1% w/w of protein) were added as the plasticizer and cross-linker, respectively. The mixture was stirred for 1 h at 40°C to allow enzymatic cross-linking. Subsequently, LEO was incorporated into the mixture at concentrations of 0%, 2.5%, and 5.0% (w/v) using Tween 80 as an emulsifier at a fixed surfactant-to-oil ratio of 1:4 (v/v) to obtain the final film-forming solutions (FFS) prior to degassing.

### Film packaging setup and indicator attachment

To prepare the films, the BPA indicators (2 × 2 cm) were first placed at the bottom of non-stick casting plates (dimensions: 20 cm × 30 cm), followed by the addition of 50 mL of degassed film FFS. This configuration ensured that the indicators remained on the film surface destined to face the package headspace above the chicken meat. The cast films were then dried at 35 ± 2°C for 24 h and conditioned at 25 ± 1°C and 50 ± 5% RH for 48 h prior to characterization. For the reference group using commercial polyethylene (PE) films, a food-grade adhesive was used to attach the BPA indicators (2 × 2 cm) onto the film surface. The indicators were positioned to face the package headspace above the chicken meat, maintaining an identical setup to the composite film groups.

### Application of smart packaging on broiler breast meat samples

Fresh broiler breasts meat (∼24 h postmortem) were obtained from a local slaughterhouse and transported at 4°C within 30 min. Samples (150 ± 10 g) were placed in polypropylene trays and assigned to four treatments: (1) Commercial overwrap PE film, (2) Gelatin-Sodium alginate without LEO (Control), (3) Gelatin-Sodium alginate containing 2.5% LEO (LEO 2.5%), and (4) Gelatin-Sodium alginate containing 5.0% LEO (LEO 5%). Packaged samples were stored at 4 ± 1°C, and quality parameters were evaluated on days 0, 1, 3, 5, and 7.

### Physical and physicochemical characterization of films

Film thickness was measured at six random positions using a digital micrometer (293-832-30, Mitutoyo, Japan). Surface color (L*, a*, b*) was evaluated using a portable colorimeter (HunterLab ColorQuest XE, model CQX382). The Swelling Index (SI**)** was determined according to Elhadef et al. (2024); 2 cm x 2 cm film specimens were weighed (W_i_), immersed in 30 mL of distilled water for 2 min, blotted dry, and immediately re-weighed (W_f_). SI was calculated using the following equation:

$$SI\;\left(\%\right)=\left(\frac{W_{f}-\;W_{i}}{W_{i}}\right)\times \;100$$

Where W_i_ (g) and W_f_ (g) represent the initial and final weights of the film, respectively.

The mechanical properties of the films, including tensile strength (TS), elongation at break (EAB) and stress–strain curves were evaluated using a TA.XT Plus texture analyzer (Stable Micro Systems, UK) with a 5 N load cell and a crosshead speed of 15 mm/min, according to the method described by Dang et al. (2024) with slight modifications. Prior to testing, specimens (30 mm x 150 mm) were equilibrated at 25°C and 50% RH for 48 h. Specimens were clamped vertically between two mechanical grips with an initial gauge length of 30 mm. TS (MPa) was calculated by dividing the maximum tensile force (N) at film rupture by the cross-sectional area (m²). EAB (%) was calculated as the percentage increase in film length relative to the initial gauge length. Additionally, the continuous load-deformation data were recorded for each specimen to construct the characteristic stress–strain curves, which visually illustrated the dynamic deformation and fracture behavior of the developed films.

Light transmission (200–800 nm) was measured via UV-Vis spectrophotometry (Jasco V-630, Japan). Film opacity was calculated as follows: O = A_600_/L, where A_600_ is the absorbance at 600 nm and L is the thickness (mm) (Kanatt, 2020).

Biodegradability was assessed following Silva et al. (2020). Film specimens (3 cm x 3 cm) were buried 4 cm deep in loam soil (pH 7.2 ± 0.2). Soil moisture was maintained by adding 20 mL of distilled water every 48 h. After 15, 30, 45, and 60 days, samples were retrieved, cleaned, and dried at 60°C to determine weight loss (W_L_):

$$W_{L}\left(\%\right)=\frac{\left(W_{0}-\;W_{t}\right)}{W_{0}}\times \;100$$

Where W_0_ is the initial weight and W_t_ is the weight after the designated degradation interval.

### Evaluation of packaging efficiency on broiler breast meat quality

Microbial quality was evaluated by homogenizing 10 g of meat with 90 mL of 0.1% sterile peptone water (Oxoid, UK) for 2 min. Serial dilutions were prepared and inoculated as follows: Total Viable Count (TVC) on PCA (incubated at 30°C for 72 h; ISO 4833-1); *E. coli* on EMB agar (incubated at 37°C for 24 h) and *S. aureus* on Baird-Parker agar with egg yolk tellurite (incubated at 37°C for 48 h; ISO 6888-1). Additionally, *Pseudomonas* spp. was enumerated on Pseudomonas CFC selective agar incubated at 25°C for 48 h according to ISO 13720. Results were expressed as log CFU/g.

Meat pH was determined using a calibrated digital pH meter (SevenCompact S220, Mettler-Toledo, Switzerland) after homogenizing 5 g of sample with 45 mL of distilled water (1:10 w/v). Drip loss was calculated based on the weight difference (Takma and Korel, 2019):

$$Drip\;loss\left(\%\right)=\frac{\left(W_{i}-\;W_{t}\right)}{W_{i}}\times \;100$$

Where W_i_ is the initial weight of the meat (g) at Day 0 and W_t_ is the weight of the sample at the specific sampling time (t).

Surface color (L*, a*, b*) was measured using a Chroma Meter (CR-350, Konica Minolta, Japan) after the samples rested at 4°C for 30 min to facilitate blooming. Measurements were performed in triplicate at six random locations per sample.

TVB-N content was determined via semi-micro steam distillation (Balamatsia et al., 2006). Homogenized samples (10 g) were mixed with 1 g of MgO, and distillation was performed using an automatic Kjeldahl analyzer (K9840, Hanon, China). The distillate was collected in 2% boric acid and titrated with 0.01 M HCl. Results were expressed as mg/100 g.

Lipid oxidation was quantified using the TBARS extraction method (Barbosa-Pereira et al., 2014). Briefly, 10 g of meat was homogenized with 10% TCA, filtered, and reacted with 0.02 M TBA at 95°C for 35 min. Absorbance was measured at 532 nm (Shimadzu, Japan) against a TMP standard curve. Results were expressed as mg MDA/kg of meat.

### Color evaluation of freshness indicators during storage

Color alterations of the freshness indicator were monitored using the CIE L*a*b* system via a portable colorimeter (HunterLab ColorQuest XE, model CQX382). The device was positioned directly over the packaged film to record the L*, a*, and b* values in triplicate throughout the storage period. The overall color change (ΔE) was subsequently determined using the following formula

$$\Delta E\;=\;\sqrt{\left\{{\left(L^{*}-\;L_{0}^{*}\right)}^{2}\pm \;{\left(a^{*}-\;a_{0}^{*}\right)}^{2}\pm \;{\left(b^{*}-\;b_{0}^{*}\right)}^{2}\right\}}$$

Where *L*_0_*, *a*_0_*, and *b*_0_* are the initial L*, a*, and b* values of the freshness indicator.

### Statistical analysis

Data were derived from six independent experimental units per treatment (n = 6) and expressed as mean ± standard deviation. Statistical analysis was performed via one-way ANOVA for a completely randomized design using the General Linear Model (GLM) procedure of SPSS (Version 26.0, IBM Corp., Armonk, NY, USA). Significant differences between means were determined using Duncan’s multiple range test at a confidence level of *p* < 0.05. To visualize multivariate relationships, Principal Component Analysis (PCA) was performed in R (Version 4.3.1) using 12 standardized variables: meat pH, drip loss, TBARS, TVB-N, TVC, and color parameters (L*, a*, b*) for both meat and film. All variables were mean-centered and scaled prior to analysis, with results presented as a PCA biplot to illustrate correlations between quality indicators and treatments over time.

## Results and discussion

### Physical and mechanical properties of composite films

#### Film thickness and structural modulation

The incorporation of lemongrass essential oil (LEO) significantly modulated the physical dimensions of the gelatin/alginate composite films in a concentration-dependent manner (*p* < 0.001; Table 1). Thickness increased progressively with LEO loading; the 5% LEO formulation was thickest (0.18 ± 0.01 mm), significantly exceeding the Control (0.12 ± 0.01 mm) and commercial PE film (0.01 ± 0.00 mm). These values are consistent with the established range for biopolymer-based packaging (0.159–0.384 mm), which critically governs mechanical durability and mass transfer resistance (Kanatt, 2020; Shaikh et al., 2019; Shanbhag et al., 2023). In this study, the emulsified LEO droplets acted as molecular spacers, disrupting the dense intermolecular cross-linking between gelatin and sodium alginate chains, thereby provoking matrix expansion (Dashipour et al., 2015; Khezrian and Shahbazi, 2018). This structural disruption increases the free volume within the polymeric lattice, effectively inflating the film’s cross-sectional profile (Haghighi et al., 2019; Chu et al., 2019). Functionally, the synergistic effects of increased thickness and the presence of dispersed oil phases extend the 'tortuous path' for gas diffusion. Functionally, this expanded matrix structure facilitates the sustained release of bioactive volatiles, such as citral (Rhim and Ng, 2007; Realini and Marcos, 2014). By maintaining a continuous supply of antioxidant volatiles, the 5.0% LEO film is designed to mitigate lipid peroxidation primary driver of quality loss in PUFA-rich broiler meat. Consequently, this dual-action mechanism ensures the stability of the broiler breast meat appearance by preventing oxidative discoloration (metmyoglobin formation, albeit at low concentrations, and lipid-linked browning), thereby extending the functional longevity of the active packaging. Similar matrix expansion trends have been noted in gelatin films using oregano and rosemary oils (Tongnuanchan and Benjakul, 2012; Souza et al., 2018).

**Table 1:** Comparative analysis of physical and mechanical properties of gelatin/alginate composite films enriched with lemongrass essential oil (LEO) and commercial PE film.

Treatments						
Parameter	Commercial-PE film	Control	**LEO** 2.5%	**LEO** 5%	SEM	P-value
Film thickness (mm)	0.01±0.00^D^	0.12±0.01^C^	0.15±0.01^B^	0.18±0.01^A^	0.003	<0.001
Swelling capacity (%)	0.93±0.07^D^	76.67±0.22^A^	72.90±1.06^B^	69.71±0.11^C^	0.21	<0.001
Solubility (%)	0.96±0.06^D^	62.14±0.99^A^	54.67±0.96^B^	45.84±0.62^C^	0.46	<0.001
Tensile strength (TS) MPa	12.70±0.07^A^	11.36±1.38^B^	10.92±0.09^BC^	8.66±0.08^C^	0.65	<0.001
Elongation at break (EAB)	81.44±2.01^A^	58.14±2.48^C^	60.39±2.44^C^	62.32±2.69^B^	2.72	<0.001
Opacity Value	1.02 ± 0.11^C^	2.04 ± 0.12^B^	2.23 ± 0.16^AB^	2.35±0.21^A^	0.001	<0.001
Color						
L*	52.82±0.09^A^	48.38±0.41^B^	47.29±0.05^C^	45.09±0.48^D^	0.22	<0.001
a*	0.56±0.04^A^	−0.17±0.01^D^	−0.06±0.02^C^	0.16±0.01^B^	0.01	<0.001
b*	−1.47±0.03^D^	1.20±0.02^C^	2.21±0.02^B^	2.69±0.04^A^	0.03	<0.001
ΔE	5.20±0.33^A^	0.00±0.00^D^	0.90±0.08^C^	3.63±0.10^B^	0.07	<0.001

Values are presented as mean ± standard error (n = 6). Different superscript letters (A–D, within the same row indicate significant differences (*p* < 0.05)).

#### Optical properties and light barrier capacity

The visual appearance of the commercial PE film, control gelatin/alginate film, and composite films incorporated with 2.5% and 5% LEO is shown in Fig. 2. The optical characteristics of the gelatin/alginate composite films, including CIELAB parameters (L*, a*, b*) and spectral transmittance, were significantly modulated by LEO concentration (*p* < 0.001; Table 1). Lightness (L*) decreased while yellowness (b*) increased progressively; the 5.0% LEO film exhibited the lowest L* (45.09 ± 0.48) and a significant total color difference (ΔE = 3.63) relative to the control. This shift toward the yellow coordinate (b*) is attributed to the intrinsic chromatic properties of LEO’s bioactive constituents, particularly citral and terpenoids, which naturally possess a yellowish hue (Siracusa et al., 2018; Liu et al., 2020). Furthermore, the incorporation of LEO into the biopolymer matrix often leads to a more intense yellow hue as the oil concentration increases, a phenomenon commonly observed in lemongrass essential oil-based films (Ahmad et al., 2026). This intensification is likely driven by the light-scattering effect at the interfaces of the lipid droplets dispersed within the gelatin/alginate composite network, which alters the optical reflectance of the film (Mendes et al., 2020). Despite these chromatic shifts, the films retained sufficient functional transparency, ensuring that the internal colorimetric freshness indicators remain clearly visible for consumer monitoring, which represents a vital feature for "smart" poultry packaging (Bajić et al., 2019; Kraśniewska and Gniewosz, 2025).

**Fig. 2:**
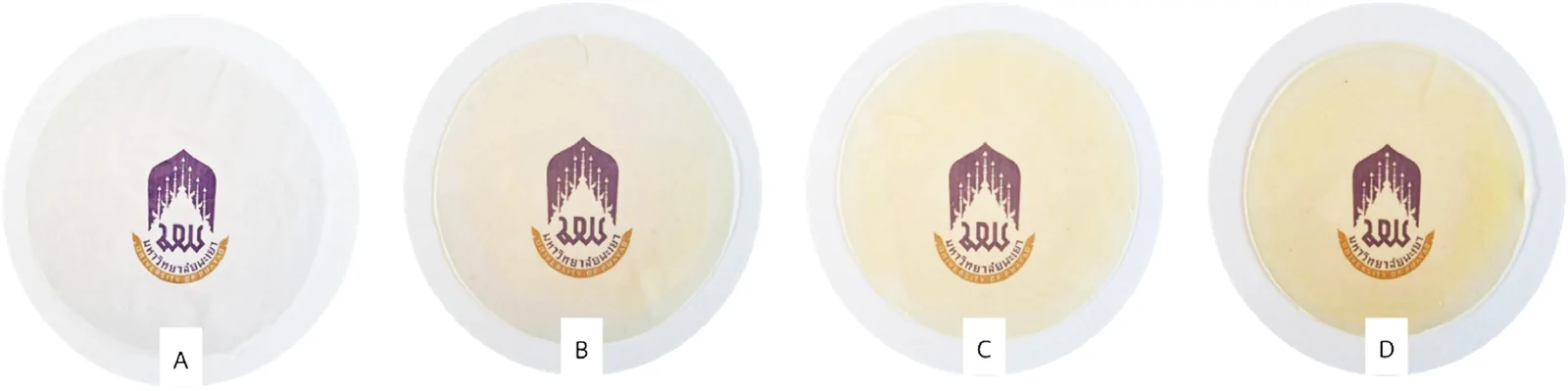
Visual appearance of Commercial-PE film -PE film (A), Control film: 100% Gelatin-Sodium alginate + 0% LEO (B), Gelatin-Sodium alginate+ 2.5% LEO (C), Gelatin-Sodium alginate + 5% LEO (D).

Regarding light barrier capacity, LEO significantly enhanced UV shielding compared to commercial PE film (*p* < 0.05; Fig. 3). The 5.0% LEO formulation exhibited the lowest UV transmittance (66.83% at 200 nm), while visible light transmission (600 nm) dropped from 77.89% (control) to 72.89%. Opacity values (2.04–2.35) rose due to light scattering at the interfaces of hydrophobic LEO microdroplets and the hydrophilic matrix (Noori et al., 2018; Marzlan et al., 2022; Kraśniewska and Gniewosz, 2025). Consequently, the LEO-functionalized film acts as a potent UV-shield, where the conjugated structure of citral absorbs radiation while oil droplets induce light scattering (Roy and Rhim, 2021). In poultry science, this UV-shielding is critical; it mitigates light-induced lipid oxidation, which is the primary catalyst for off-flavor development and ancillary pigment fading in broiler breast meat during retail display (Waga and Nakade, 2025; Zhang et al., 2025). Such enhanced photostability, consistent with findings for other essential oils, works alongside LEO’s antimicrobial properties to preserve meat sensory attributes during refrigeration (Kanatt, 2020; Raj et al., 2024).

**Fig. 3:**
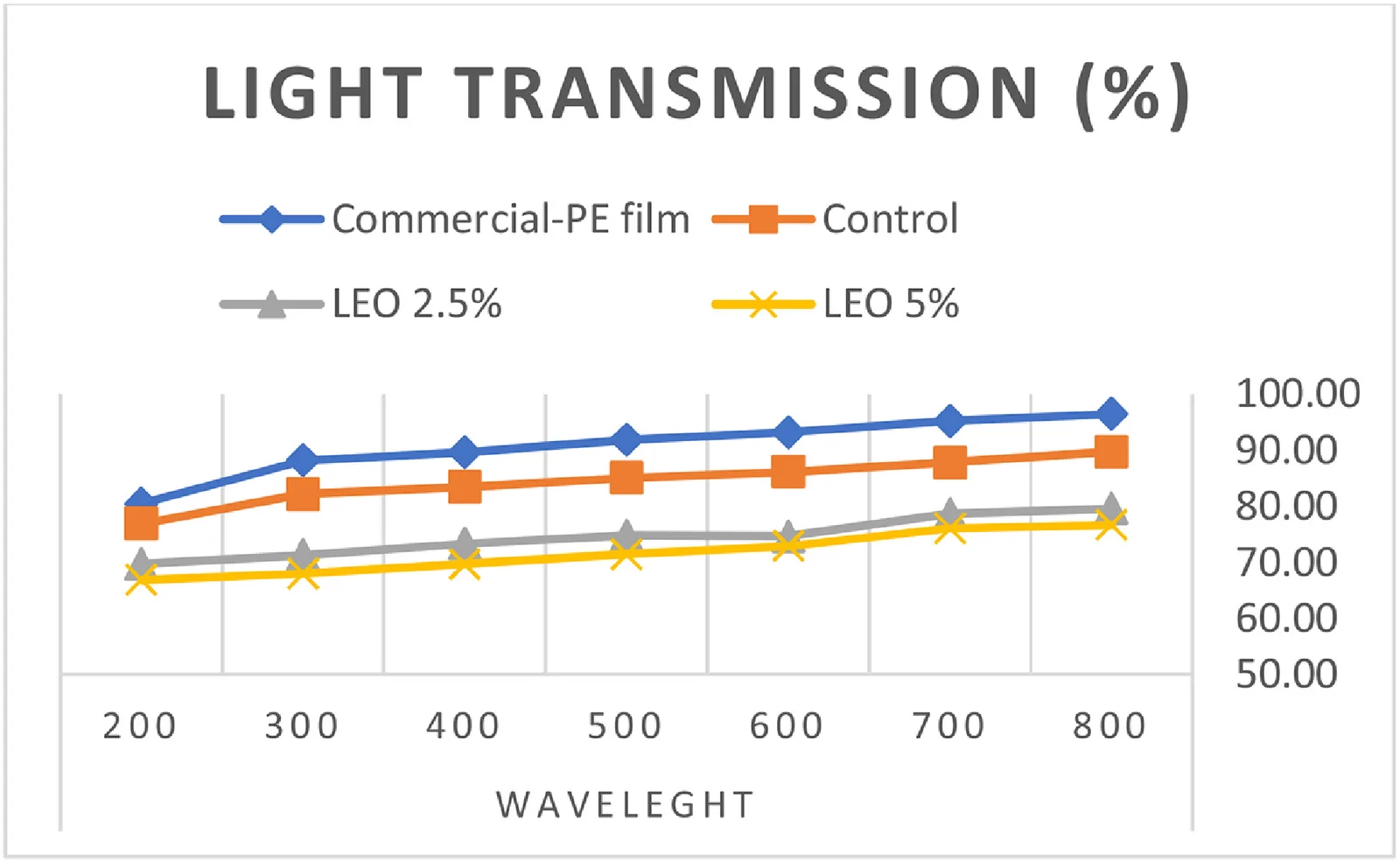
UV-visible light transmittance (%) of gelatin/alginate composite films incorporated with different concentrations of lemongrass essential oil (LEO) compared to commercial polyethylene (PE) film across the 200–800 nm wavelength range.

### Swelling capacity and water solubility

Water solubility and swelling capacity are critical determinants of the structural stability of biopolymer films, particularly when applied to high-moisture foods like broiler breast. As shown in Table 1, both parameters decreased significantly as LEO concentration increased (*p* < 0.001). Compared to the Control, the LEO 5% formulation reduced water solubility by 26% and limited the swelling index to 69.71%, demonstrating superior hydrophobic stability. This reduction in water affinity, despite the increase in free volume mentioned in the structural modulation section, is primarily attributed to the predominant hydrophobic effect of LEO’s bioactive constituents, such as citral. hese non-polar segments restrict water molecules from interacting with the hydrophilic hydroxyl groups of the gelatin/alginate matrix (Yadav et al., 2021). Consistent with Kraśniewska and Gniewosz (2025) and Daza et al. (2023), the dispersed LEO droplets promote hydrophobic interactions and molecular entanglements within the polymeric lattice, effectively limiting chain relaxation and subsequent water association. Additionally, the TGase-mediated cross-linking establishes covalent ε-(γ-glutamyl) lysine bones, which reinforce the protein architecture and create a network highly resistant to osmotic disintegration (Ahammed et al., 2021). Ultimately, LEO-incorporated films maintain their structural integrity and barrier efficacy when exposed to meat exudates (drip loss), preventing premature dissolution of the active packaging (Realini and Marcos, 2014; Ahmad et al., 2026). Achieving this balance between hydrophobicity and structural flexibility is essential for developing active packaging with high antimicrobial and barrier performance (Pavli et al., 2018; Knani et al., 2018; Kumar et al., 2025).

### Mechanical properties

The mechanical performance of the developed films, summarized in Table 1, was significantly modulated by LEO concentration (*p* < 0.001). A dose-dependent decline in tensile strength (TS), from 11.36 ± 1.38 MPa (Control) to 8.66 ± 0.08 MPa (5.0% LEO), resulted from the plasticizing effect of LEO. This reduction in tensile resistance is clearly reflected in the typical stress–strain curves (Fig. 5), where the peak stress of the LEO 5% film was the lowest. Mechanistically, the oil microdroplets disrupt the intermolecular hydrogen bonding within the gelatin/alginate network, replacing strong polymer-polymer interactions with weaker oil-polymer associations (Daza et al., 2023). Conversely, Elongation at Break (EAB) significantly improved, reaching 64.32% in the 5.0% LEO treatment. This behavior signifies a classic plasticizing effect; the hydrophobic LEO droplets act as internal lubricants, accommodating themselves between adjacent gelatin and alginate chains. This integration increases the free volume and chain mobility within the matrix, allowing the polymer chains to slide past one another more easily under mechanical stress, which shifts the rupture point toward higher strain values (Fig. 5). Furthermore, this LEO-induced plasticization acts synergistically with the TGase-mediated ε-(γ-glutamyl) lysine crosslinking establishes a reinforced yet flexible network (Zubair and Ullah, 2020). While this structural expansion typically facilitates water penetration, the intrinsic hydrophobicity of LEO's constituents (e.g., citral) effectively counteracts this effect by reducing the film's overall affinity for water. Therefore, LEO exerts a dual role: enhancing the film's ductility (EAB) through plasticization while simultaneously maintaining hydrophobic stability, as evidenced by the significant reduction in swelling capacity**.** Although the lipid phase reduces the overall rigidity (TS), the TGase-induced covalent bonds provide the necessary cohesive strength necessary to prevent premature structural failure during stretching. Regarding practical application, such heightened ductility is vital for poultry packaging; it enables the material to adhere closely to the irregular geometry of broiler breast meat while preventing structural rupture. This close contact is essential for maintaining the integrity of the protective microenvironment and ensuring the sustained release of LEO volatiles across the meat surface throughout the cold chain (Song et al., 2018; Marzlan et al., 2022).

### Biodegradability analysis

The environmental compatibility of packaging materials is a pivotal factor in mitigating the ecological burden of non-degradable plastic waste. As demonstrated in Fig. 5, all gelatin/alginate composite films exhibited significantly higher biodegradation rates compared to the Commercial-PE film over the 60-day soil burial period (*p* < 0.001). While the Commercial-PE remained virtually intact with a negligible weight loss of only 1.18%, the control gelatin/alginate composite film reached 80.32% degradation. This stark contrast underscores the superior eco-friendly profile of biopolymer matrices over conventional synthetic polymers (Mathew et al., 2019). The incorporation of LEO exerted a concentration-dependent influence on the biodegradation kinetics. By day 60, the biodegradation percentage decreased from 80.32% (Control) to 70.99% in the 5.0% LEO formulation. This retardation can be attributed to two primary mechanisms. First, the hydrophobic nature of LEO components, such as citral, limits water diffusion into the film matrix, thereby delaying the initial swelling and subsequent enzymatic hydrolysis by soil microorganisms (Yadav et al., 2021). Second, the inherent antimicrobial properties of LEO likely suppress the metabolic activity of soil-borne bacteria and fungi, reducing the secretion of extracellular enzymes (e.g., proteases) required for polymer breakdown (Solanki et al., 2022). Furthermore, the use of transglutaminase (TGase) in this study creates covalent cross-links within the protein network, resulting in a more resilient and enzymatically resistant structure compared to non-crosslinked biopolymers (Mirpoor et al., 2022). These findings are consistent with previous reports suggesting that hydrophobic functional groups and stable cross-links can enhance the structural longevity of films against microbial attack during the initial stages of burial (Mathew et al., 2025). Despite the slight reduction in degradation rate induced by LEO loading, all biopolymer treatments achieved over 70% degradation within 60 days. This performance confirms that the developed proactive-diagnostic films not only provide functional benefits for broiler meat preservation but also align with SDG 12 (Responsible Consumption and Production) and SDG 13 (Climate Action) by offering a sustainable end-of-life alternative to petroleum-based packaging, effectively closing the loop in the poultry supply chain.

### Physicochemical and microbiological quality of chilled chicken meat

#### pH value and TVB-N correlation

Muscle pH and Total Volatile Basic Nitrogen (TVB-N) are definitive indices of poultry spoilage, as their elevation directly reflects the degree of microbial protein degradation and subsequent amine accumulation. In this study, a progressive and highly correlated rise in both parameters was observed throughout the 7-d storage period (Fig. 7). Initially, all samples exhibited a narrow pH range (5.95–5.97). However, by day 7, the pH of Commercial-PE samples rose sharply to 6.68 ± 0.02, signaling advanced spoilage due to accumulation of alkaline metabolites. In contrast, the 5.0% LEO film significantly stabilized the muscle pH at 6.12 ± 0.01 (*p* < 0.05), effectively maintaining values below the critical threshold for fresh poultry throughout the storage period. This pH stabilization is intrinsically linked to the TVB-N results. While the Commercial-PE samples exceeded the established spoilage limit (25 mg N/100 g) reaching 27.47 mg N/100 g by day 7, the 5.0% LEO treatment remained well within the fresh limit at 15.77 mg N/100 g. The application of active packaging can significantly delay the accumulation of Total Volatile Basic Nitrogen (TVB-N). Previous studies have demonstrated the efficacy of active packaging in mitigating TVB-N accumulation; for instance, Dirpan et al. (2022) reported that garlic extract-infused paper extended the freshness of beef up to 12 days, compared to only 6 days in the control. Similarly, Ghasemi et al. (2024) observed that NLC-Savory EO nanocomposite coatings effectively maintained TVB-N levels below the 25 mg N/100 g threshold throughout 12 days of refrigerated storage. The underlying biochemical mechanism is attributed to the potent antimicrobial action of LEO’s primary bioactive terpenoids, particularly citral. These compounds effectively suppress the proliferation of proteolytic psychrotrophs, such as *Pseudomonas* spp., thereby inhibiting the oxidative deamination of non-protein nitrogenous compounds (Yazgan et al., 2019; Xiao et al., 2024). This reduction in microbial metabolic activity subsequently prevents the accumulation of alkaline metabolites, such as ammonia (NH_₃_) and biogenic amines, which are responsible for both the pH rise and the development of off-odors (Ramezani et al., 2015; Ghollasi-Mood et al., 2016). Furthermore, the TGase-mediated cross-linking within the gelatin/alginate matrix facilitates a controlled-release system. By reinforcing the polymeric network, TGase ensured that a lethal concentration of LEO volatiles is sustained at the meat–packaging interface over time, preventing rapid surface alkalinization. These findings align with Kocatepe et al. (2025), who reported that microbial transglutaminase enhances the functional longevity of essential oil-based films. Consequently, the synergistic effect of active LEO release and the structural integrity of the TGase-crosslinked matrix successfully preserved the chemical freshness of the broiler breast meat, extending its shelf-life compared to conventional petroleum-based packaging.

#### Drip loss dynamics

Drip loss, a critical determinant of poultry quality, reflects the myofibrillar matrix's ability to retain intrinsic water against postmortem biochemical alterations. As shown in Fig. 7, no significant differences were observed among treatments on day 0 (*p* > 0.05), yet treatment-specific effects became pronounced as storage progressed. The Commercial-PE group exhibited the highest drip loss (14.17±2.20%) by day 7, whereas the 5.0% LEO film significantly curtailed fluid exudation to 6.66% (*p* < 0.05). This marked reduction suggests that LEO-functionalized films effectively stabilized the muscle protein architecture through a multi-modal mechanism. Primarily, the bioactive terpenoids in LEO, particularly citral, act as a potent radical scavenger that mitigates protein carbonylation and the denaturation of myofibrillar proteins, thereby preserving the capillary forces required to retain moisture (Nurul Syahida et al., 2020; Ahmad et al., 2026). Furthermore, the film’s antimicrobial action, evidenced by lower TPC and TVB-N values, suppressed the secretion of microbial extracellular proteases. The inhibition prevents the structural breakdown of the perimysium and endomysium, which leads to myofibrillar shrinkage and subsequent fluid leakage (Huff-Lonergan and Lonergan, 2016; Purslow, 2020). Additionally, the TGase-reinforced gelatin/alginate matrix provides a superior physical barrier that limits the mass transfer of moisture at the meat–packaging interface. The balanced hydrophobicity of the LEO-incorporated film, as discussed in the solubility analysis, helps maintain a stable microenvironment that minimizes osmotic pressure changes on the meat surface. Consequently, the 5.0% LEO-loaded film functions as a robust bioactive barrier that preserves the structural integrity and succulence of broiler breast meat throughout the cold chain (Lawrence et al., 2015; Zeng and Ertbjerg, 2017).

#### Total plate count (TPC) and shelf-life extension

The primary objective of using active packaging in meat products is to reduce the Total Plate Count (TPC) and inhibit spoilage-related microflora. Bacterial growth is the most critical factor in determining the shelf-life of fresh meat products. In this study, TPC results indicated that the Commercial-PE group approached the microbiological spoilage threshold (7.0 log CFU/g) by day 7, reaching 6.67±0.02 log CFU/g. Conversely, the 5.0% LEO film significantly inhibited microbial growth, maintaining a substantially lower count of 5.67±0.02 log CFU/g (*p* < 0.05) (Table 2). This concentration-dependent antimicrobial efficacy is primarily attributed to the hydrophobic nature of LEO constituents, particularly citral. These bioactive compounds partition into the lipid bilayer of bacterial membranes, disrupting the lipopolysaccharide structure and increasing membrane permeability. This structural compromise leads to the irreversible leakage of essential intracellular constituents, such as ions and ATP, resulting in the loss of cellular homeostasis and eventual cell death (Burt, 2004; Faheem et al., 2022). Furthermore, the TGase-crosslinked gelatin/alginate matrix serves as an effective carrier that modulates the volatility of LEO. By preventing rapid evaporation, the polymer network ensures a sustained release of bioactive terpenoids onto the meat surface, maintaining a lethal concentration at the meat–packaging interface throughout storage (Ahamad et al., 2021). This synergistic interaction between the structural matrix and the active essential oil not only ensures microbiological safety but also validates the film’s potential as an active packaging system capable of extending the shelf-life of fresh broiler breast meat beyond the standard duration.

**Table 2:** Impact of LEO-incorporated gelatin/alginate composite films on the microbial growth of chicken breast meat during 7 days of refrigerated storage (4 ± 2°C).

**Parameter**	**Days**	**Treatments**					
		**Commercial-PE film**	**Control**	**LEO 2.5%**	**LEO 5%**	**SEM**	**P-value**
**TPC** **log CFU/g**	**0**	3.40 ± 0.04^A^^e^	3.26 ± 0.04 Be	3.09 ± 0.04^C^^e^	2.88 ± 0.04^D^^e^	0.035	<0.001
	**1**	3.79 ± 0.03^A^^d^	3.56 ± 0.03 Bd	3.35 ± 0.03^C^^d^	3.18 ± 0.03^D^^d^	0.027	<0.001
	**3**	4.48 ± 0.06 A^c^	4.42 ± 0.06 Bc	4.30 ± 0.06 Bc	4.10 ± 0.06^C^^c^	0.049	<0.001
	**5**	5.69 ± 0.03 Ab	4.94 ± 0.03^B^^b^	4.86 ± 0.03^B^^b^	4.67 ± 0.03^C^^b^	0.024	<0.001
	**7**	6.67 ± 0.02 Aa	6.25 ± 0.02 Ba	5.92 ± 0.02^C^^a^	5.67 ± 0.02^D^^a^	0.018	<0.001

Note: Values are presented as mean ± standard error (n = 6).

A-C Means within the same row (Treatment) with different letters are significantly different (*p* < 0.05).

a-e Means within the same column (Day) with different letters are significantly different (*p* < 0.05).

#### Dynamics of meat color attributes

The surface color of broiler breast meat is a primary determinant of consumer acceptance and perceived freshness. In this study, the interaction between pigment stability and lipid oxidation was significantly modulated by the LEO-incorporated gelatin/alginate composite films (Table 3). Throughout the 7-day storage period, a significant decline in L* (lightness) and b* (yellowness) was observed across all treatments (*p* < 0.05). This downward trend typically signifies the advancement of pigment oxidation and the gradual formation of metmyoglobin (MetMb), which results in a duller, discolored appearance (Mancini and Hunt, 2005; Han et al., 2024). Notably, the 5.0% LEO group exhibited superior color retention, maintaining the highest a* value by day 7 (*p* < 0.001) compared to the Commercial-PE and Control groups. This preservation of the desirable pinkish hue is attributed to the potent antioxidant micro-environment facilitated by the active film. The bioactive terpenes in LEO, specifically citral and geraniol, act as hydrogen donors that effectively scavenge peroxy radicals. This mechanism shields the heme iron (Fe^2±^) and the globin polypeptide chain from oxidative attack, thereby delaying the onset of pigment discoloration (Kumar et al., 2017). These findings align with Zhang et al. (2025) and Zhai et al. (2019), confirming that essential oil-loaded films extend the color stability of fresh meat by delaying the transition of oxymyoglobin to the pro-oxidative ferric metmyoglobin (Fe^3±^) state.

**Table 3:** Effects of LEO-incorporated gelatin/alginate composite films on the meat color (L*, a*, b*) of breast meat during 7 days of chilled storage.

**Parameter**	**Days**	**Treatments**					
		**Commercial-PE film**	**Control**	**LEO 2.5%**	**LEO 5%**	**SEM**	**P-value**
L*	0	40.54±0.16^a^	40.53±0.31^a^	40.52±0.43^a^	40.52±0.07^a^	0.08	0.99
	1	40.16±0.13^a^	40.24±0.37^a^	40.30±0.22^a^	40.49±0.17^a^	0.07	0.446
	3	39.96±0.07^B^^b^	40.08±0.11^A^^a^^b^	40.16±0.15^A^^a^^b^	40.26±0.07^A^^a^	0.03	0.042
	5	38.30±0.52^B^^b^	39.17±0.05^A^^a^^b^	39.51±0.23^A^^a^^b^	39.64±0.34^A^^a^^b^	0.1	0.005
	7	37.23±0.06^B^^c^	38.25±0.23^A^^b^	38.38±0.08^A^^b^	38.58±0.39^A^^b^	0.07	<0.001
a*	0	1.28±0.11^d^	1.33±0.11^c^	1.35±0.11^b^	1.40±0.11^b^	0.03	0.231
	1	1.35±0.04^B^^c^^d^	1.38±0.05^A^^B^^b^^c^	1.41±0.08^A^^B^^b^	1.47±0.25^A^^b^	0.04	0.122
	3	1.37±0.04^C^^b^^c^	1.43±0.04^A^^B^^b^^c^	1.48±0.02^B^^C^^b^	1.53±0.07^A^^b^	0.01	0.01
	5	1.47±0.03^B^^a^^b^	1.48±0.08^B^^a^^b^	1.55±0.03^A^^B^^b^	1.64±0.05^A^^a^^b^	0.01	0.015
	7	1.53±0.02^D^^a^	1.60±0.03^C^^a^	1.70±0.03^B^^a^	1.75±0.01^A^^a^	0.01	<0.001
b*	0	7.25±0.07^a^	7.46±0.12^a^	7.37±0.16^a^	7.33±0.16^a^	0.04	0.322
	1	6.82±0.05^a^^b^	6.79±0.21^a^^b^	6.82±0.05^b^	6.79±0.21^a^^b^	0.04	0.98
	3	6.45±0.38^b^	6.58±0.52^b^	6.52±0.26^b^	6.55±0.50^a^^b^	0.12	0.985
	5	6.82±0.05^a^^b^	6.79±0.21^a^^b^	6.80±0.05^b^	6.79±0.21^a^^b^	0.04	0.989
	7	5.56±0.02^B^^c^	5.65±0.42^B^^c^	5.56±0.02^B^^c^	6.45±0.38^A^^b^	0.08	0.012

Note: Values are presented as mean ± standard error (n = 6).

A-C Means within the same row (Treatment) with different letters are significantly different (*p* < 0.05).

a-e Means within the same column (Day) with different letters are significantly different (*p* < 0.05).

#### Mitigation of lipid oxidation

The stabilization of surface color in muscle foods is intrinsically linked to the mitigation of lipid oxidation. As presented in Table 3, TBARS values increased significantly (*p* < 0.05) across all groups during storage; however, the LEO-incorporated films demonstrated a robust, concentration-dependent inhibitory effect. By day 7, the 5% LEO treatment effectively maintained malondialdehyde (MDA) levels at 1.32 ± 0.10 mg MDA/kg, remaining well below the widely accepted sensory spoilage threshold of 2.0 mg MDA/kg (Domínguez et al., 2019; Jung et al., 2025). In contrast, the Commercial-PE group exceeded this limit by Day 5 (2.09 ± 0.16 mg MDA/kg). The active film extended the oxidative shelf-life by approximately 40%, significantly delaying the onset of oxidative rancidity. The superior performance of the 5% LEO treatment suggests that higher antioxidant loading facilitates a sustained release of volatile bioactive constituents, such as the terpenoids myrcene and citral, from the gelatin/alginate matrix. This observation aligns with reports by Adilah and Hanani (2016) regarding the application of *Morinda citrifolia* oil in active packaging. The efficacy of these systems is often modulated by complex interactions between the film polymer and the botanical extract (Martucci et al., 2015), where specific essential oil components undergo molecular binding within the matrix, governing their migration kinetics for radical scavenging activities (Găboreanu and Marinaş, 2023). This controlled-release mechanism prevents premature volatilization, ensuring prolonged protection of polyunsaturated fatty acids (PUFAs), which are particularly susceptible in poultry muscle. Furthermore, the LEO-incorporated films initiated a protective cascade preserving the muscle’s structural integrity. Secondary lipid oxidation products, specifically MDA and 4-hydroxynonenal (4-HNE), are potent catalysts for myoglobin oxidation and the induction of protein carbonylation (Ma et al., 2017). Such oxidative modifications typically lead to the loss of myofibrillar protein solubility and a subsequent decrease in Water-Holding Capacity (WHC) (Zeng and Ertbjerg, 2017). Our observation of significantly higher a* values in LEO-treated samples on day 7 is attributed to enhanced moisture retention within the muscle matrix and the prevention of pigment degradation. By suppressing TBARS accumulation, the 5% LEO film indirectly preserves the myofibrillar lattice, thereby reducing drip loss and preventing the dull, dark appearance characteristic of advanced oxidative deterioration in poultry. These results confirm that LEO-enriched films function as a multifunctional active packaging system, stabilizing the chemical, structural, and visual attributes of broiler breast meat.

#### Antimicrobial efficacy and mechanistic action of LEO-active films

The integration of lemongrass essential oil (LEO) into gelatin/alginate composite films exerted a robust, concentration-dependent inhibitory effect against both *E. coli* and *S. aureus* (Table 4). These species were selected as representative models due to their frequent association with foodborne outbreaks and poultry spoilage (Takma and Korel, 2019). A pivotal finding was the higher susceptibility of *S. aureus* (1.42 log CFU/g reduction) compared to *E. coli* (0.55 log CFU/g) by day 7, this differential sensitivity is intrinsically linked to the distinct cell envelope architectures of Gram-positive and Gram-negative bacteria. While the lipopolysaccharide-rich outer membrane of *E. coli* serves as a highly selective hydrophilic barrier that restricts the diffusion of hydrophobic terpenes, the porous peptidoglycan layer of *S. aureus* allows lipophilic citral molecules to readily partition into the cytoplasmic membrane. This induces a fatal bioenergetic failure through the dissipation of the proton motive force (PMF) and subsequent leakage of essential intracellular metabolites, such as ions and ATP (Ma et al., 2017; Kumar et al., 2025). The enhanced antimicrobial performance is attributed to the uniform distribution of LEO droplets within the biopolymer matrix. This structural arrangement significantly increases the interfacial surface area and bioavailability of active constituents at the meat–film interface (Acevedo-Fani et al., 2015). These fine droplets are physically entrapped within the crosslinked fish gelatin and sodium alginate network. This dense polymeric lattice, induced by transglutaminase, functions as a tortuous path that governs the controlled release of the active compounds. This structural integration effectively anchors the LEO and modulates its diffusion kinetics through a dual-action mechanism involving both direct interfacial contact and a vapor phase inhibitory effect. Volatile citral is gradually released from this hydrogel matrix into the micro-headspace between the film and the surface of meat, ensuring a sustained lethal concentration that can reach and penetrate microbial membranes even in the irregular crevices and pores of the broiler breast meat surface where direct contact is limited (Rojas-Graü et al., 2007). The sustained antimicrobial activity through day 7 highlights the efficacy of this active film as a viable substitute for synthetic preservatives. Notably, the significant inhibition of *E. coli* observed at the 5% LEO concentration suggests that high-density bioactive loading can partially destabilize the Gram-negative cell envelope by inducing surface distortion and neutralizing the protective functions of the lipopolysaccharide layer (Kieling et al., 2021). Furthermore, the absence of *Bacillus cereus* and *Salmonella* spp. across all groups indicates high initial raw material quality and stringent hygienic handling, thereby allowing the active packaging to selectively target and suppress primary spoilage indicators. Consequently, this synergistic system ensures superior microbiological stabilization and extends the shelf-life of chilled poultry meat while satisfying the demand for natural ingredients (Kocatepe et al., 2025).

**Table 4:** Changes in microbial counts (*E. coli and S. aureus*) of chicken meat packaged with gelatin/alginate composite films incorporated with lemongrass essential oil (LEO) during refrigerated storage.

Microbial count	Storage	Treatment				
(Log CFU/g)	(Day)	Control	LEO 2.5%	LEO 5%	SEM	p-value
*E. coli*	0	2.26±0.24^e^	2.10±0.17 e	2.15±0.21^c^	0.068	0.494
	1	2.78±0.23^A^^d^	2.54±0.27^A^^B^^d^	2.30±0.30^B^^c^	0.104	0.105
	3	3.30±0.12^A^^c^	3.10±0.15^A^^B^^c^	2.85±0.25^B^^b^	0.084	0.02
	5	4.61±0.05^A^^b^	4.45±0.04^B^^b^	4.28±0.07^C^^a^	0.051	0.006
	7	5.10±0.06^A^^a^	4.75±0.08^B^^a^	4.55±0.12^B^^a^	0.084	0.011
*S. aureus*	0	2.26±0.24^d^	2.10±0.17^c^	2.00±0.00^c^	0.068	0.475
	1	2.50±0.19^A^^c^	2.26±0.24^B^^b^	2.10±0.17^B^^b^	0.082	0.008
	3	3.29±0.14^A^^b^	2.36±0.10^B^^a^	2.26±0.24^B^^a^	0.171	0.002
	5	4.34±0.02^A^^a^	4.17±0.05^A^^B^^a^	3.94±0.15^B^^a^	0.063	0.03
	7	5.41±0.02^A^^a^	4.24±0.05^B^^a^	3.99±0.07^C^^a^	0.062	0.002

Note: Values are presented as mean ± standard error (n = 6).

A-C Means within the same row (Treatment) with different letters are significantly different (*p* < 0.05).

a-e Means within the same column (Day) with different letters are significantly different (*p* < 0.05). Pathogens including *B. cereus* and *Salmonella spp*. were not detected (< 1 Log CFU/g) throughout the study.

### Integrative quality assessment via PCA: a multidimensional spoilage map

Principal Component Analysis (PCA) explained 85.5% of the total variance, with PC1 (74.0%) representing the primary axis of spoilage progression (Fig. 8). PC1 showed strong positive loadings for TBARS (0.976), TVB-N (0.976), and TPC (0.951), while exhibiting an inverse correlation with meat a* (redness) and film b* (yellowness). This clear separation confirms that microbial proliferation and lipid oxidation are the synergistic drivers of pigment degradation and overall quality loss. PC2 (11.5%) was primarily influenced by drip loss (0.922), effectively serving as an indicator of muscle structural integrity. The PCA biplot (Fig. 9) revealed a distinct temporal migration of all samples toward the spoilage quadrant (characterized by high TPC, TVB-N, and TBARS) (Fig. 11); however, the rate of migration varied significantly among treatments. Control and Commercial-PE samples exhibited a rapid rightward drift along PC1 (reaching values up to ±7.36 by day 7), indicating a severe accelerated collapse in chemical and microbiological quality. Conversely, the 5% LEO treatment maintained the highest degree of proximity to the initial "fresh-meat region," terminating at a remarkably lower PC1 score of ±0.89 by day 7. his superior stability stems from the sustained and controlled release of citral from the TGase-crosslinked matrix, which effectively neutralizes multiple spoilage pathways (Zhang et al., 2025). Ultimately, the 5.0% LEO-incorporated films successfully preserve the meat’s multidimensional quality profile near its baseline state, providing a robust, clean-label solution for enhancing poultry safety and shelf-life.

### Dynamic real-time monitoring of broiler freshness indicator

The efficacy of the pH-sensitive indicator films in monitoring the real-time freshness of refrigerated broiler breast meat demonstrated a significant correlation with biochemical spoilage indices. Fig. 4 demonstrates the pH-dependent color transitions (halochromic behavior) of butterfly pea (Clitoria ternatea L.) extract within the pH range of 2–12. Fig. 6, Fig. 8). This color transition synchronized with the rapid accumulation of TVB-N (27.47 mg N/100g) and meat pH (6.68) shown in Table 2, both of which clearly exceeded the freshness threshold for poultry meat (Sornchuer et al., 2020). (ΔE) To quantify these visual shifts and evaluate the sensitivity of freshness indicator, the total color difference (ΔE) was calculated relative to Day 0 (Fig. 12). According to colorimetric principles in intelligent food packaging system, a ΔE value exceeding 2.0 indicates a color variation perceptible to the human eye, whereas a greater than 5.0 indicates a definitive, macroscopic color shift typically driven by a micro-environmental pH alteration of 0.5–1.0 unit (Wu et al., 2024). On Day 1, the film incorporated with 5% LEO exhibited the most rapid color acceleration, crossing the visual detection limit with a ΔE of 2.21, whereas the Control film displayed negligible color variation (ΔE=1.28). By Day 7, the ΔE values across all treatments increased significantly (P<0.001) (ΔE>5.0). The Control film reached a final ΔE of 5.71, which was slightly above the macroscopic perceptual threshold, which synchronized with the accumulation of TVB-N and the rise in meat pH, both of which exceeded the freshness threshold for poultry meat (Sornchuer et al., 2020). Conversely, the 5% LEO film demonstrated a highly pronounced and accelerated colorimetric response, drastically exceeding the visual threshold by Day 5 (ΔE=16.68) and reaching a final ΔE of 19.34 by Day 7.

**Fig. 4:**
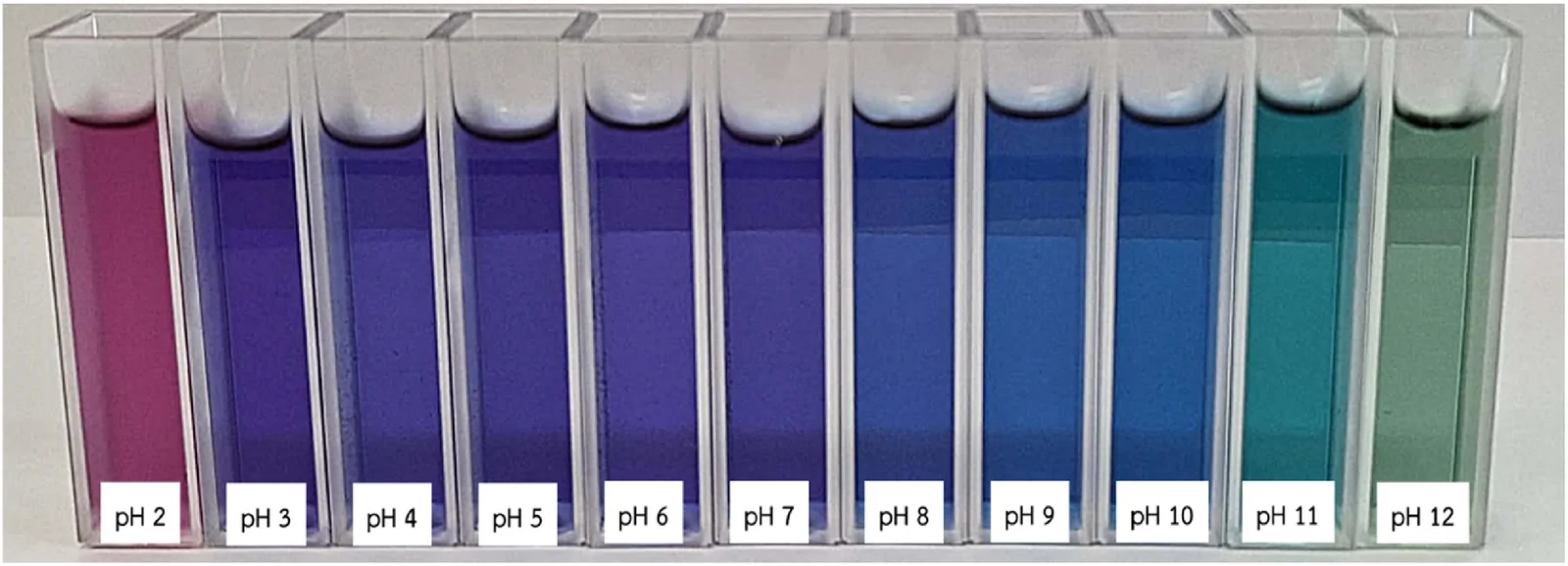
Demonstrates the pH-dependent color transitions (halochromic behavior) of butterfly pea (*Clitoria ternatea* L.) extract within the pH range of 2–12.

**Fig. 5:**
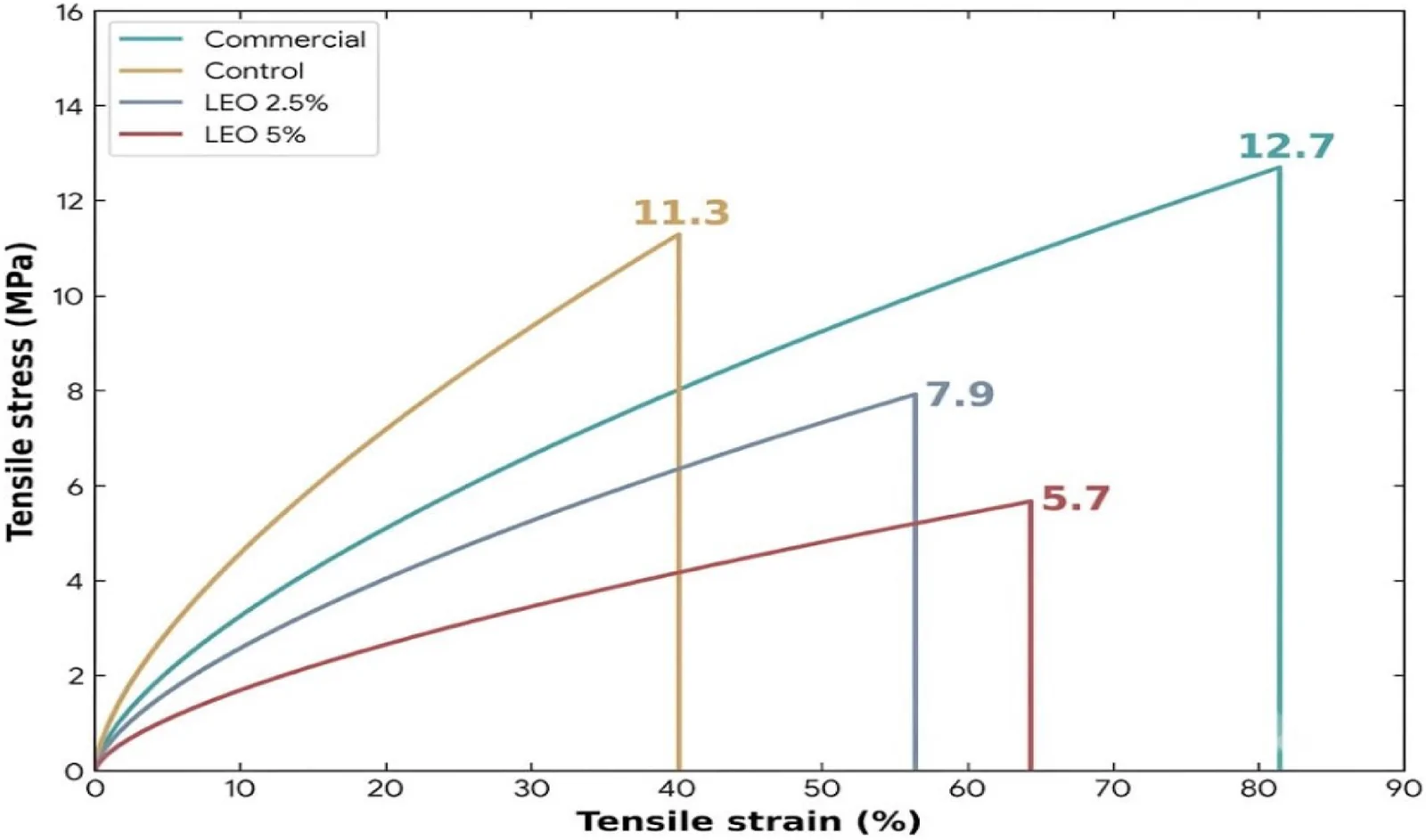
Typical tensile stress–strain curves of the developed films at various LEO concentrations.

**Fig. 6:**
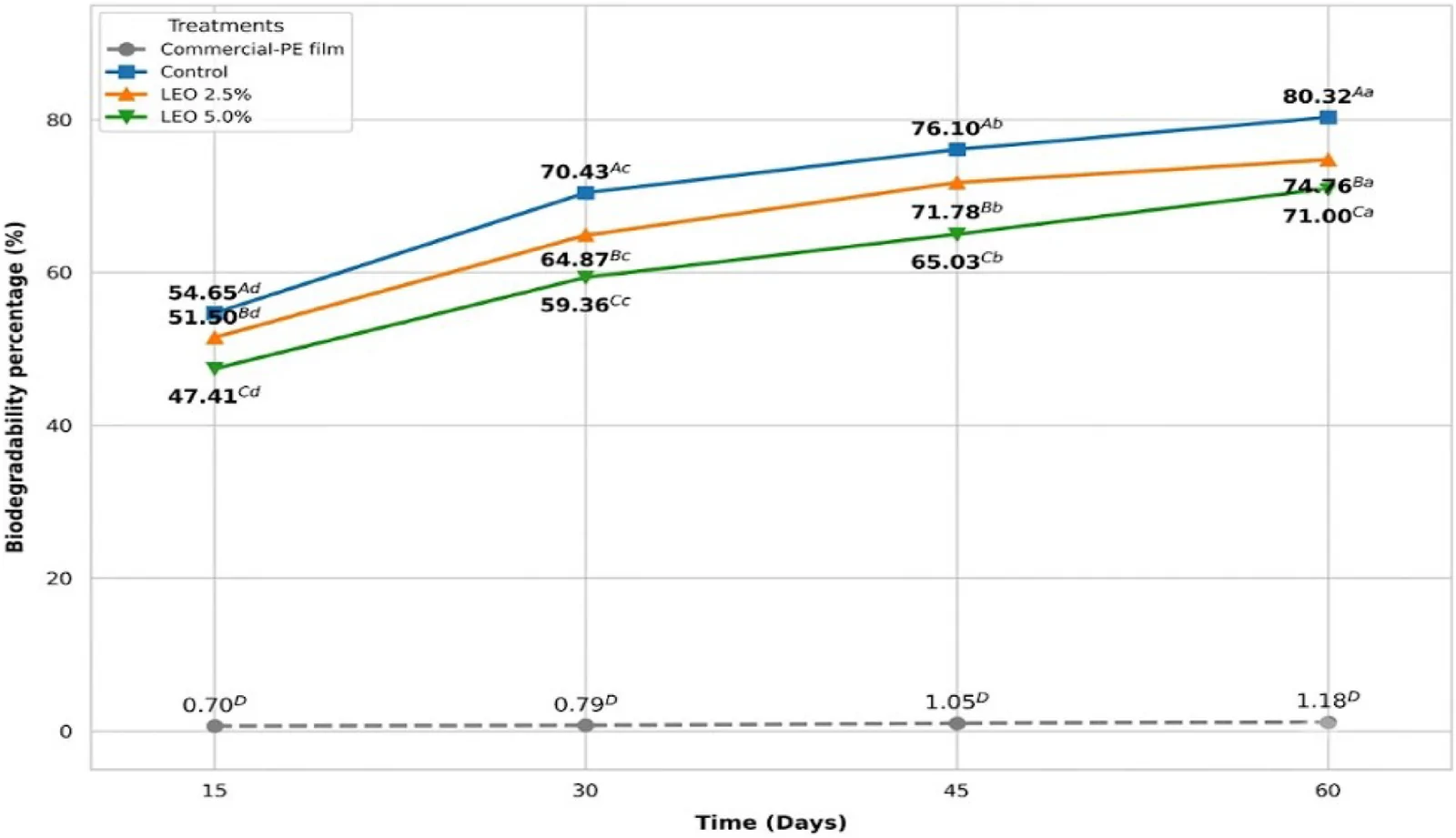
Biodegradability percentage of gelatin/alginate composite films incorporated with varying levels of lemongrass (*Cymbopogon citratus*) essential oil compared to commercial polyethylene film over a 60-day soil burial period. ^A–D^: Different uppercase superscripts indicate significant differences between treatments within the same storage day (*p* < 0.05). ^a–e^: Different lowercase superscripts indicate significant differences across storage days within the same treatment (*p* < 0.05).

**Fig. 7:**
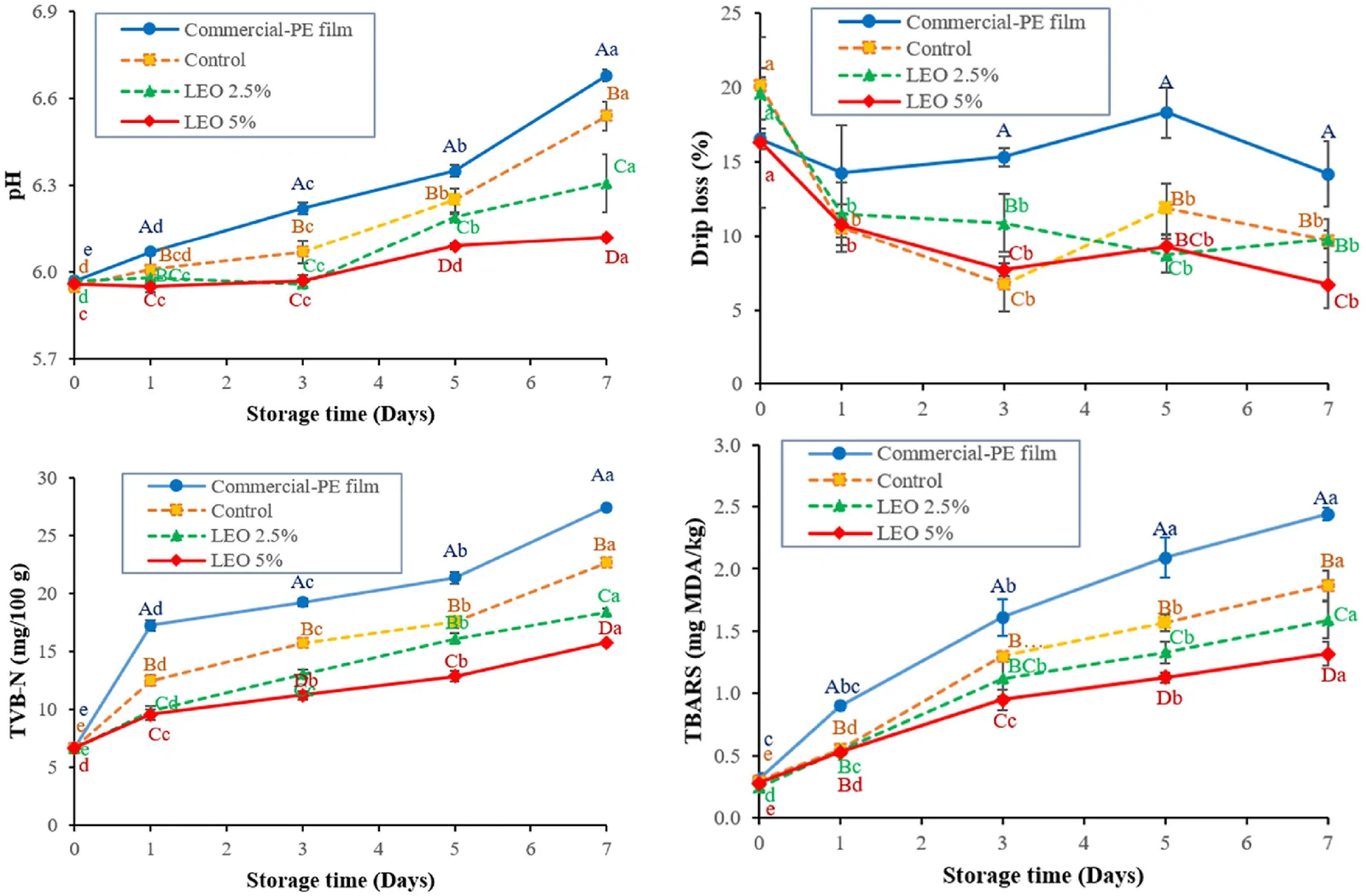
Effects LEO-incorporated gelatin/alginate composite films on the pH (A), drip loss (B), total volatile basic nitrogen (TVB-N) (C), and thiobarbituric acid reactive substances (TBARS) (D) of breast meat during 7 days of refrigerated (4°C) storage. Error bars represent standard deviations of the means. ^A–D^: Different uppercase superscripts indicate significant differences between treatments within the same storage day (*p* < 0.05). ^a–e^: Different lowercase superscripts indicate significant differences across storage days within the same treatment (*p* < 0.05).

**Fig. 8:**
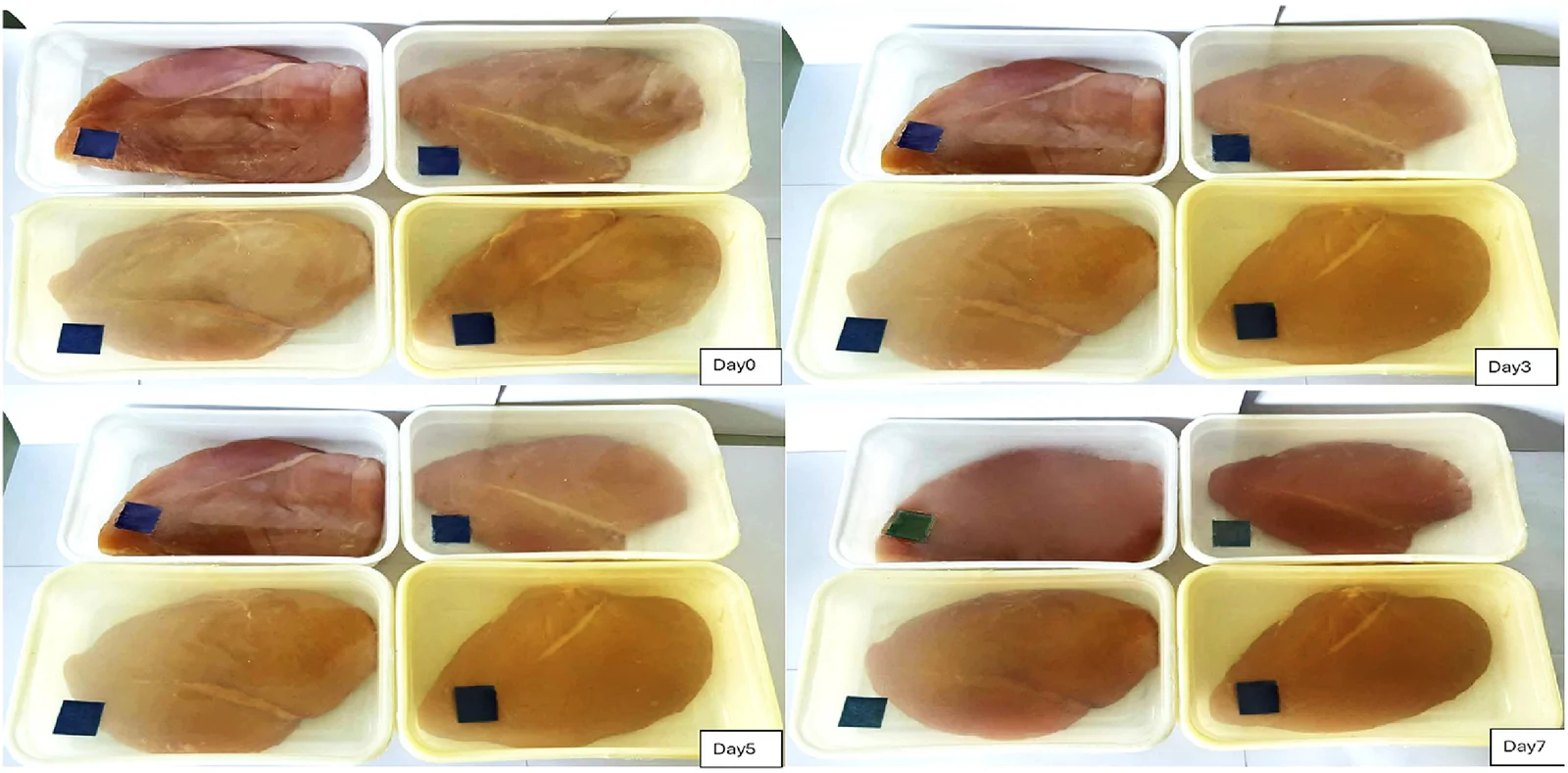
Chicken breast fillet storage samples with smart packaging during storage at 4°C for 7 days.

**Fig. 9:**
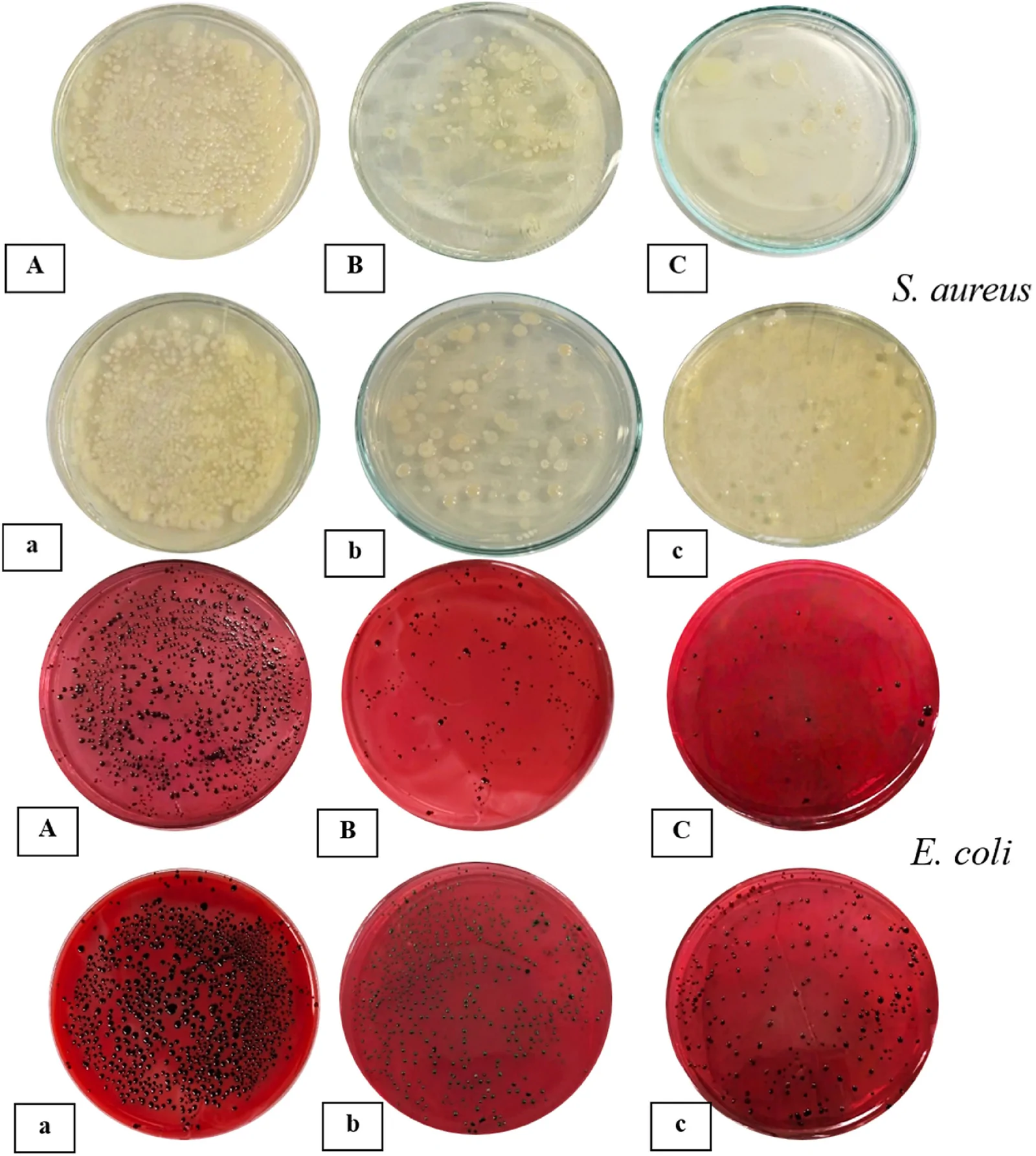
*E. coli* and *Staphylococcus aureus* growth on chicken meat packaged with various films during storage at $4\pm 2^{\circ }C$ after 3 days (A–C) and 7 days (a–c). (A, a) Control (0% LEO), (B, b) 2.5% LEO and (C, c) 5% LEO.

**Fig. 10:**
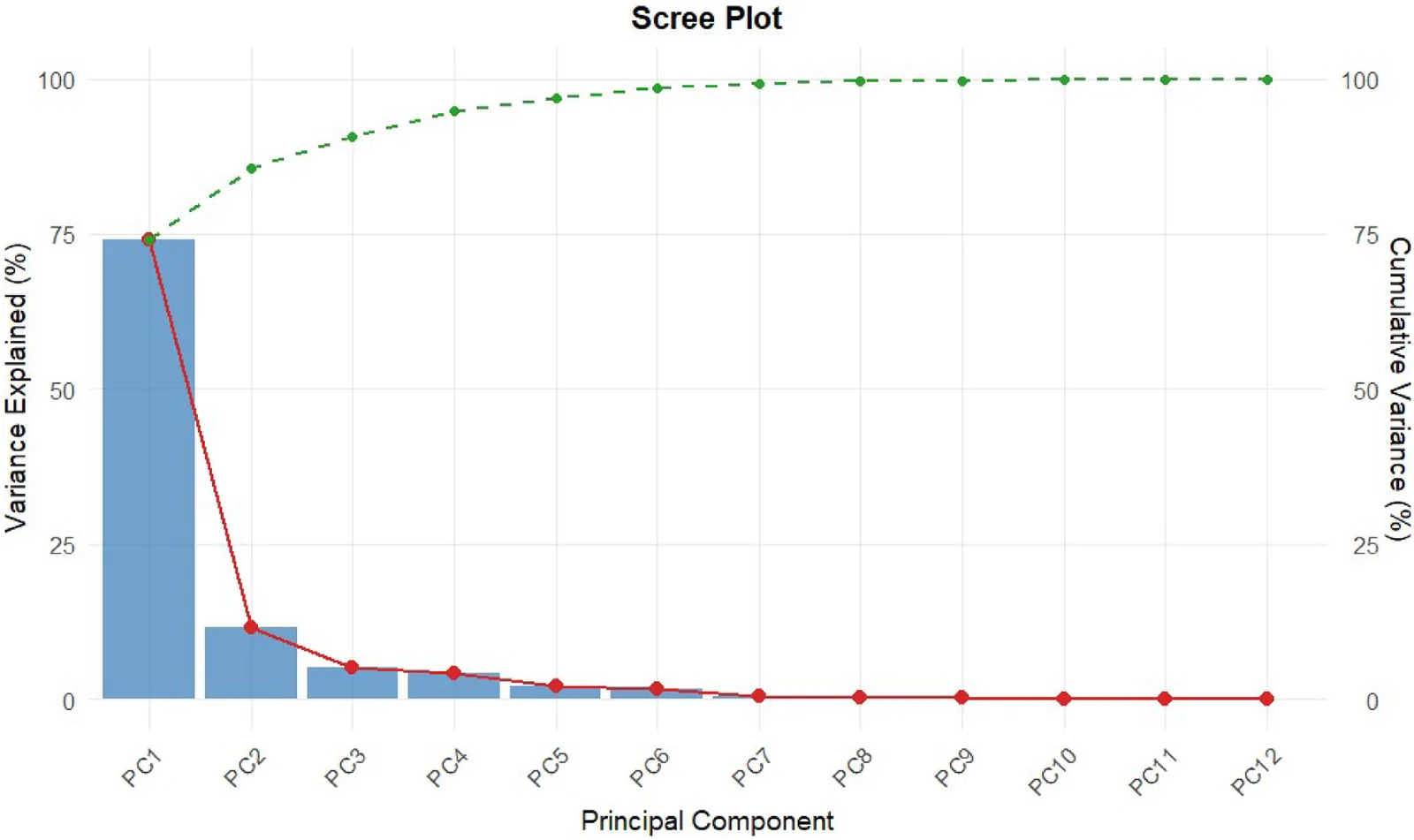
Scree plot showing the variance explained by each principal component.

**Fig. 11:**
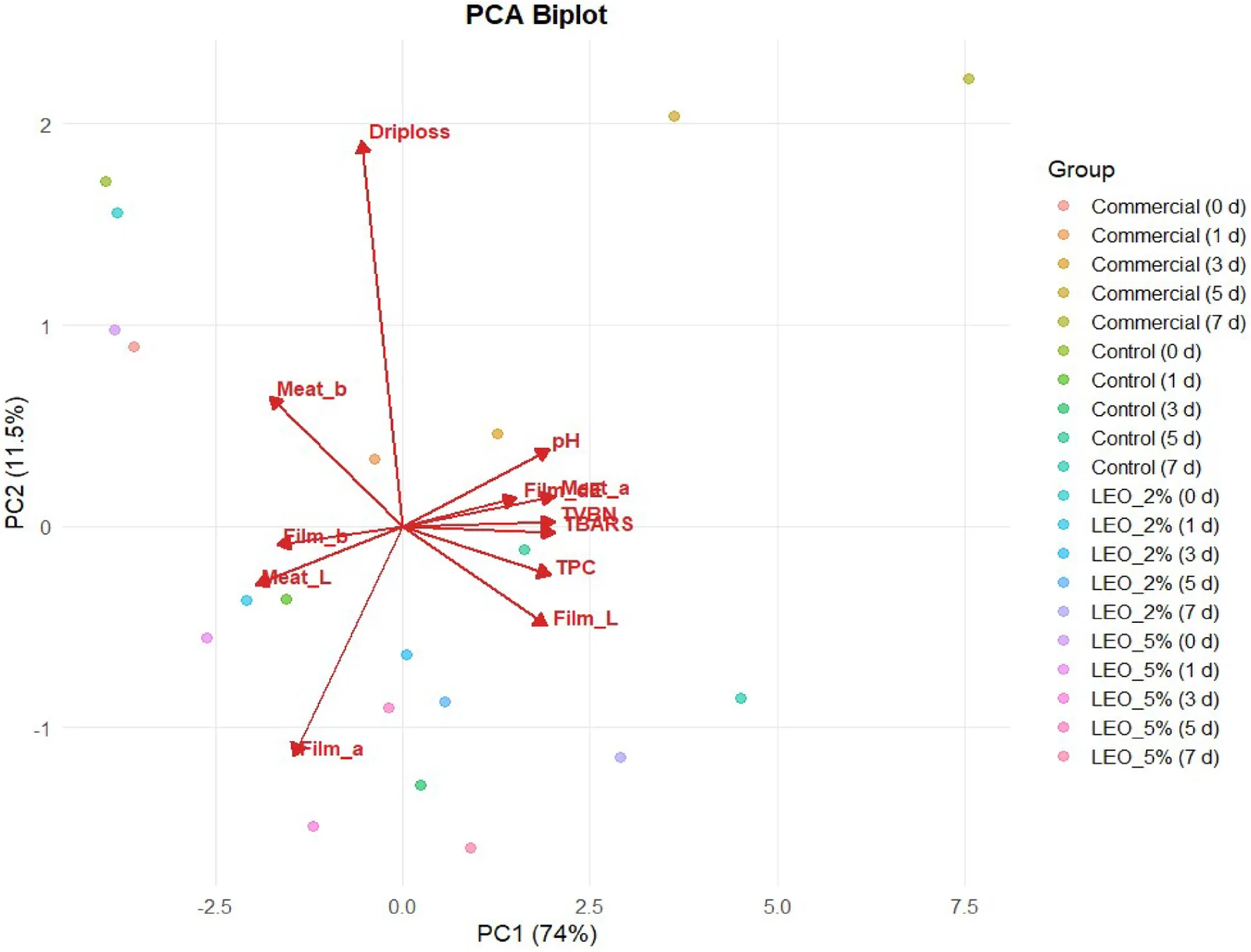
PCA biplot showing the separation of treatments over storage time along PC1 and PC2.

**Fig. 12:**
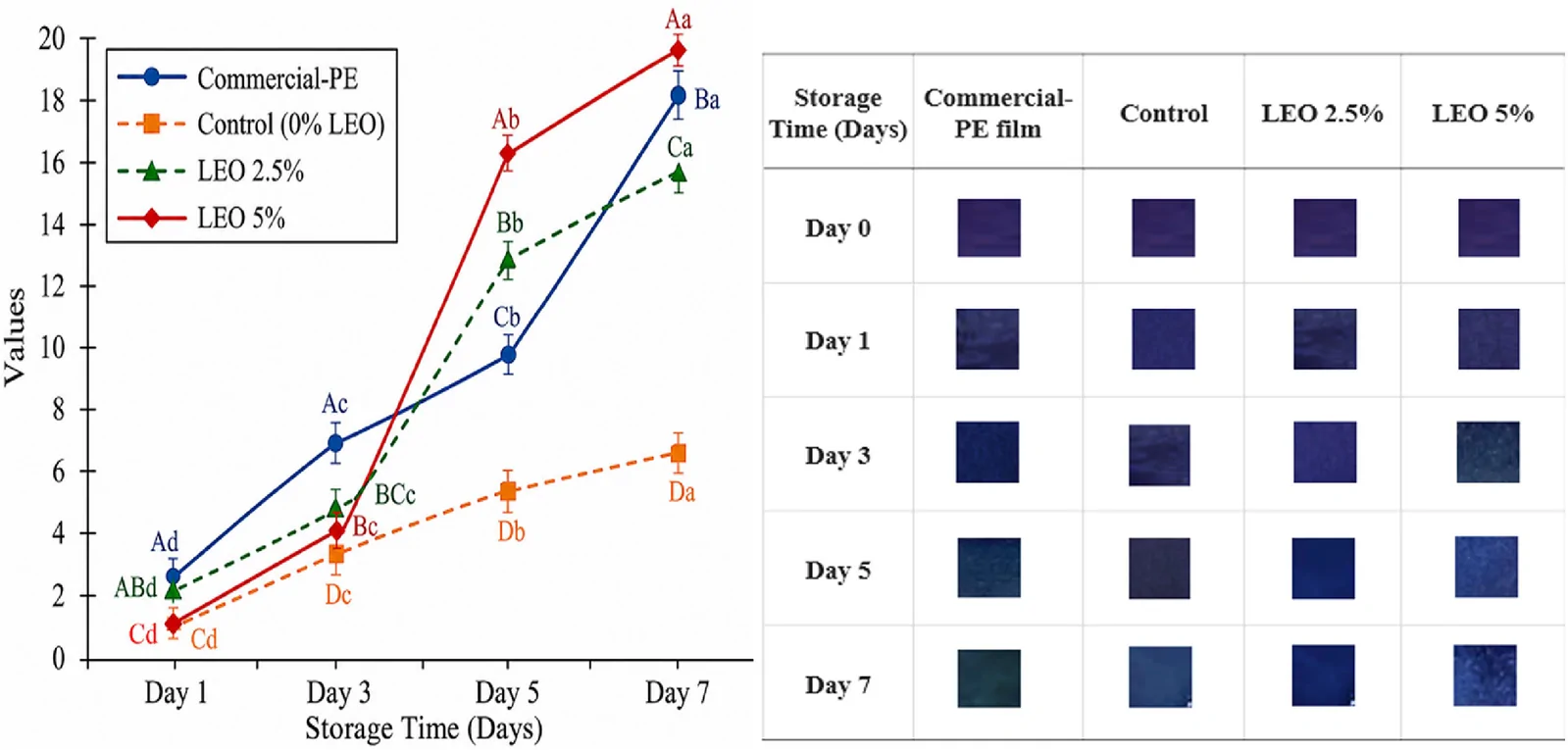
Total color difference (ΔE) and color transition of the freshness indicator films during storage of chicken meat at 4°C for 7 days. Commercial-PE: Commercial polyethylene film; Control: Gelatin-Sodium alginate + 0% LEO; LEO 2.5%: Gelatin-Sodium alginate+ 2.5% LEO; LEO 5%: Gelatin-Sodium alginate + 5% LEO. ^A–D^: Different uppercase superscripts indicate significant differences between treatments within the same storage day (*p* < 0.05). ^a–e^: Different lowercase superscripts indicate significant differences across storage days within the same treatment (*p* < 0.05).

Despite the substantial ΔE values recorded by the instrumental colorimetry, a discrepancy was observed between the instrumental sensitivity and actual visual perception, as the physical transition toward a distinct green or yellow hue remained subtle to the naked eye. This phenomenon can be primarily attributed to a significant darkening of the indicator during storage; the lightness (L*) parameter decreased drastically from 43.90 to 28.71, imparting a pronounced darkening effect. Concurrently, the yellowness/blueness (b*) parameter coordinate shifted deeply into negative domain, declining from 0.945 to −10.24 (Table S1). Consequently, this profound loss of lightness and intense shift toward blue masked the newly emerging hue, rendering the macroscopic color change less visually distinct to the consumer, despite the high variance recorded instrumentally.

This characteristic halochromic transition is mediated by the structural transformation of delphinidin derivatives from the butterfly pea extract. As proteolytic bacteria metabolize muscle amino acids, they release volatile basic nitrogen (TVB-N) compounds, such as ammonia (NH_3_) and trimethylamine (N(CH_3_)_3_), into the package headspace (Albertos et al., 2017; Romruen et al., 2024). The accumulation of these alkaline volatiles induces the deprotonation of anthocyanins, shifting their molecular structure from the stable flavylium cation (purple/blue) to the quinoidal base, and ultimately toward the chalcone form (greenish yellow) under progressive alkalization (Boonsiriwit et al., 2021; Li et al., 2026). However, in high-moisture environments typical of poultry packaging, free anthocyanins are highly susceptible to nucleophilic attack by water molecules, which accelerates the irreversible degradation of the colorless chalcone form and causes color fading Xiong et al., 2024). This phenomenon was explicitly observed in the indicator system attached to the unencapsulated gelatin/alginate composite film group (commercial PE packaging group), which exhibited a pronounced fading of color intensity by Day 7 due to direct and continuous exposure to excessive internal moisture and headspace condensation. Conversely, the sustained stability of the color parameters in the gelatin/alginate composite film without fading suggest that the encapsulation of delphinidin within the gelatin/alginate polymeric network, supplemented by the hydrophobic micro-environment of LEO, effectively shielded the anthocyanin molecules from moisture-induced degradation. Recent advancements in smart bio-polymeric indicators have highlighted that matrix density and local hydrophobicity are key determinants of volatile amine diffusion rates (Li et al., 2026).

Aligning with these insights, the incorporation of LEO appears to exerted a synergistic dual effect on the smart packaging system. On the meat surface, the antimicrobial action of LEO’s bioactive components, particularly citral, suppressed the proliferation of spoilage microflora, thereby limiting TVB-N generation and delaying meat alkalization. Concurrently, within the polymer matrix, the hydrophobic oil droplets likely disrupted the compact hydrophilic gelatin/alginate network, inducing micro-porosity. This structural modification facilitated the mass transfer of volatile compounds (Rhim and Ng, 2007; Realini and Marcos, 2014), allowing volatile alkaline amines to diffuse efficiently and interact with the embedded anthocyanins. Crucially, while micro-porosity typically risks exposing hydrophilic anthocyanins to destructive packaging moisture, the hydrophobic nature of the co-embedded LEO droplets acted as a localized moisture barrier within the polymer matrix. This synchronized protection restricted excessive water hydration of the delphinidin core, ensuring that the accelerated ΔE values primarily reflected the volatile amine accumulation rather than environmental pigment degradation. Consequently, the indicator maintained high instrumental responsiveness despite the decelerated spoilage rate of the meat.

Nevertheless, it must be acknowledged that the visual subtlety of the hue transition currently constrains immediate, naked-eye commercial monitoring by consumers. To bridge this translational gap, future investigations will focus on optimizing the visual clarity of the indicator system. Practical strategies under consideration include: (i) formulating mixed indicator profiles (e.g., co-pigmentation with structurally complementary dyes) to yield higher color contrast; (ii) designing a high-opacity white backing material within the indicator patch to amplify subtle shifts in chromaticity; and (iii) integrating the smart film with a dedicated smartphone application capable of digitally decoding the colorimetric coordinates for end-users.

## Conclusion

This study successfully developed a multifunctional, proactive-diagnostic smart packaging system by integrating lemongrass essential oil (LEO) and butterfly pea anthocyanin (BPA) into a TGase-crosslinked fish gelatin/alginate matrix. In addition to enhancing the physicochemical properties of the film, particularly its UV shielding capacity and water resistance, the incorporation of LEO exerted potent antimicrobial and antioxidant effects. These functionalities effectively disrupted the bioenergetic failure pathways of *E. coli* and *S. aureus* while retarding the oxidative cascade that leads to lipid and protein degradation in refrigerated broiler breast meat. Consequently, this active functionality maintained the meat’s structural integrity and succulence, effectively extending the microbial shelf-life by at least 3 days compared to conventional petroleum-based packaging. Simultaneously, the BPA-based sensor functioned as a reliable, non-destructive visual indicator. The distinct colorimetric transition from dark purple to blue and green was precisely synchronized with surface pH shifts and the accumulation of volatile basic nitrogen (TVB-N), reflecting the structural transformation of anthocyanins into chalcone derivatives. Furthermore, the film's excellent soil biodegradability (>70% within 60 days) underscores its potential as a sustainable alternative to petroleum-based plastics. In conclusion, this integrated packaging technology offers a scalable and eco-friendly framework to enhance food safety and consumer confidence within the poultry industry. By aligning with a circular economy and global sustainability targets (SDGs 12 and 13), this innovation provides a robust strategy for minimizing economic losses and ensuring premium quality monitoring throughout the cold chain.

## Author Contribution

Krittanon Chotprom: Designed the research protocols, conducted the experiments, collected and analyzed data, and wrote the initial manuscript. Somchart Tana: Contributed to the research design, provided research resources, and verified the experimental results. Trakul Prommajak: Conceived the study concept, supervised the research project, and revised the manuscript. Kunakorn Katsri: Participated in study conceptualization, carried out experimental work, and critically reviewed and edited the manuscript. Napatsorn Montha: Assisted in designing experimental protocols and performed data analysis. Phatthawin Setthaya: Contributed to the methodology design and assisted in drafting the manuscript. Piyanart Nuntalit: Validated the experimental findings and contributed to drafting the manuscript. Thanaporn Bunmee: Conceived and directed the research project, designed the methodology, conducted experiments, analyzed data, prepared figures, and participated in drafting and finalizing the manuscript.

## Disclosures

The authors declare that they have no known competing financial interests or personal relationships that could have appeared to influence the work reported in this paper.

## Footnotes

Supplementary material associated with this article can be found, in the online version, at doi:10.1016/j.psj.2026.107532.
