# Supplementary material for: Development of a proactive-diagnostic smart packaging system for real-time freshness monitoring and shelf-life extension of refrigerated broiler breast
Source: Poult Sci. 2026 Jul 30;105(11):107532. doi: 10.1016/j.psj.2026.107532 (PMC13629336; doi:10.1016/j.psj.2026.107532)
Supplement: Supplementary file 1 [file mmc1.docx]

**Table S1 Colorimetric response of pH-sensitive films on chicken meat during 7 days of storage at 4 ± 2 °C Picture of the indicator film during chicken storage for 8 d at 4 °C**

|  | **Commercial-PE film** | **Control** | **LEO 2.5%** | **LEO 5%** | **SEM** | **P-Value** |
| --- | --- | --- | --- | --- | --- | --- |
| **0** | **L*=45.53±0.56^ABa^** | **L*=44.13±0.50^Ba^** | **L*=46.44±0.5^Aa^** | **L*=43.90±0.54Ba** | **0.3** | **0.031** |
|  | **a*=-3.83±0.30^Aa^** | **a*=-4.47±0.43^ABa^** | **a*=-4.74±0.45^BCa^** | **a*=-5.29±0.01^Ca^** | **0.1** | **0.006** |
|  | **b*=-11.62±0.17^Dd^** | **b*=-4.95±0.13^Cc^** | **b*=-2.35±0.19^Ba^** | **b*=0.95±0.04^Aa^** | **0.04** | **<0.001** |
| **1** | **L*=43.83±0.04^BCb^** | **L*=44.47±0.07^ABa^** | **L*=44.73±1.16^Aa^** | **L*=43.54±1.18^Ca^** | **0.24** | **0.022** |
|  | **a*=-5.58±0.08^Ab^** | **a*=-5.60±0.04^Ab^** | **a*=-5.48±0.03^Ab^** | **a*=-6.07±0.25^Ab^** | **0.04** | **0.262** |
|  | **b*=-10.87±0.03^Dc^** | **b*=-5.44±0.05^Cc^** | **b*=-2.35±0.06^Ba^** | **b*=-1.20±0.21^Ab^** | **0.03** | **<0.001** |
| **3** | **L*=41.78±0.01^Cc^** | **L*=44.47±0.26^Aa^** | **L*=44.73±1.88^Aa^** | **L*=43.54±0.25^Ba^** | **0.28** | **<0.001** |
|  | **a*=-8.83±0.47^Dc^** | **a*=-8.49±0.02^Cc^** | **a*=-7.86±0.06^Bc^** | **a*=-7.31±0.03^Ac^** | **0.07** | **<0.001** |
|  | **b*=-8.37±0.17^Cb^** | **b*=-3.44±0.04^Ab^** | **b*=-4.35±0.02^Bb^** | **b*=-3.45±0.19^Ac^** | **0.04** | **<0.001** |
| **5** | **L*=40.27±0.10^Ac^** | **L*=40.47±0.24^Ab^** | **L*=34.73±0.18^Bb^** | **L*=30.29±0.12^Cb^** | **0.05** | **<0.001** |
|  | **a*=-11.83±0.10^Cd^** | **a*=-8.11±0.11^Ac^** | **a*=-9.99±1.18^Bd^** | **a*=-9.57±0.60^Bd^** | **0.19** | **<0.001** |
|  | **b*=-8.38±0.05^Db^** | **b*=-3.44±0.02^Ab^** | **b*=-4.35±0.15^Bb^** | **b*=-7.70±0.05^Cd^** | **0.02** | **<0.001** |
| **7** | **L*=38.16±0.44^Bd^** | **L*=40.62±1.08^Ab^** | **L*=32.13±0.41^Cc^** | **L*=28.71±0.42^Dc^** | **0.19** | **0.008** |
|  | **a*=-13.64±0.04^De^** | **a*=-8.75±0.04^Ad^** | **a*=-10.95±0.04^Be^** | **a*=-10.79±0.07^Be^** | **0.01** | **<0.001** |
|  | **b*=1.74±0.13^Aa^** | **b*=-1.67±0.05^Ba^** | **b*=-6.55±0.05^Cc^** | **b*=-10.24±0.04^De^** | **0.02** | **<0.001** |

**Table S1 Colorimetric response of pH-sensitive films on chicken meat during 7 days of storage at 4 ± 2 °C Picture of the indicator film during chicken storage for 8 d at 4 °C**

|  | **Commercial-PE film** | **Control** | **LEO 2.5%** | **LEO 5%** | **SEM** | **P-Value** |
| --- | --- | --- | --- | --- | --- | --- |
| **0** | **L*=45.53±0.56^ABa^** | **L*=44.13±0.50^Ba^** | **L*=46.44±0.5^Aa^** | **L*=43.90±0.54Ba** | **0.3** | **0.031** |
|  | **a*=-3.83±0.30^Aa^** | **a*=-4.47±0.43^ABa^** | **a*=-4.74±0.45^BCa^** | **a*=-5.29±0.01^Ca^** | **0.1** | **0.006** |
|  | **b*=-11.62±0.17^Dd^** | **b*=-4.95±0.13^Cc^** | **b*=-2.35±0.19^Ba^** | **b*=0.95±0.04^Aa^** | **0.04** | **<0.001** |
| **1** | **L*=43.83±0.04^BCb^** | **L*=44.47±0.07^ABa^** | **L*=44.73±1.16^Aa^** | **L*=43.54±1.18^Ca^** | **0.24** | **0.022** |
|  | **a*=-5.58±0.08^Ab^** | **a*=-5.60±0.04^Ab^** | **a*=-5.48±0.03^Ab^** | **a*=-6.07±0.25^Ab^** | **0.04** | **0.262** |
|  | **b*=-10.87±0.03^Dc^** | **b*=-5.44±0.05^Cc^** | **b*=-2.35±0.06^Ba^** | **b*=-1.20±0.21^Ab^** | **0.03** | **<0.001** |
| **3** | **L*=41.78±0.01^Cc^** | **L*=44.47±0.26^Aa^** | **L*=44.73±1.88^Aa^** | **L*=43.54±0.25^Ba^** | **0.28** | **<0.001** |
|  | **a*=-8.83±0.47^Dc^** | **a*=-8.49±0.02^Cc^** | **a*=-7.86±0.06^Bc^** | **a*=-7.31±0.03^Ac^** | **0.07** | **<0.001** |
|  | **b*=-8.37±0.17^Cb^** | **b*=-3.44±0.04^Ab^** | **b*=-4.35±0.02^Bb^** | **b*=-3.45±0.19^Ac^** | **0.04** | **<0.001** |
| **5** | **L*=40.27±0.10^Ac^** | **L*=40.47±0.24^Ab^** | **L*=34.73±0.18^Bb^** | **L*=30.29±0.12^Cb^** | **0.05** | **<0.001** |
|  | **a*=-11.83±0.10^Cd^** | **a*=-8.11±0.11^Ac^** | **a*=-9.99±1.18^Bd^** | **a*=-9.57±0.60^Bd^** | **0.19** | **<0.001** |
|  | **b*=-8.38±0.05^Db^** | **b*=-3.44±0.02^Ab^** | **b*=-4.35±0.15^Bb^** | **b*=-7.70±0.05^Cd^** | **0.02** | **<0.001** |
| **7** | **L*=38.16±0.44^Bd^** | **L*=40.62±1.08^Ab^** | **L*=32.13±0.41^Cc^** | **L*=28.71±0.42^Dc^** | **0.19** | **0.008** |
|  | **a*=-13.64±0.04^De^** | **a*=-8.75±0.04^Ad^** | **a*=-10.95±0.04^Be^** | **a*=-10.79±0.07^Be^** | **0.01** | **<0.001** |
|  | **b*=1.74±0.13^Aa^** | **b*=-1.67±0.05^Ba^** | **b*=-6.55±0.05^Cc^** | **b*=-10.24±0.04^De^** | **0.02** | **<0.001** |
